# Robust dioxin-linked metallophthalocyanine tbo topology covalent organic frameworks and their photocatalytic properties

**DOI:** 10.1093/nsr/nwae396

**Published:** 2024-11-06

**Authors:** Yucheng Jin, Qianjun Zhi, Hailong Wang, Xiaoning Zhan, Dongdong Qi, Baoqiu Yu, Xu Ding, Tianying Wang, Heyuan Liu, Mingxue Tang, Jie Liu, Jianzhuang Jiang

**Affiliations:** Beijing Key Laboratory for Science and Application of Functional Molecular and Crystalline Materials, Department of Chemistry and Chemical Engineering, School of Chemistry and Biological Engineering, University of Science and Technology Beijing, Beijing 100083, China; Beijing Key Laboratory for Science and Application of Functional Molecular and Crystalline Materials, Department of Chemistry and Chemical Engineering, School of Chemistry and Biological Engineering, University of Science and Technology Beijing, Beijing 100083, China; Beijing Key Laboratory for Science and Application of Functional Molecular and Crystalline Materials, Department of Chemistry and Chemical Engineering, School of Chemistry and Biological Engineering, University of Science and Technology Beijing, Beijing 100083, China; Beijing Key Laboratory for Science and Application of Functional Molecular and Crystalline Materials, Department of Chemistry and Chemical Engineering, School of Chemistry and Biological Engineering, University of Science and Technology Beijing, Beijing 100083, China; Beijing Key Laboratory for Science and Application of Functional Molecular and Crystalline Materials, Department of Chemistry and Chemical Engineering, School of Chemistry and Biological Engineering, University of Science and Technology Beijing, Beijing 100083, China; Beijing Key Laboratory for Science and Application of Functional Molecular and Crystalline Materials, Department of Chemistry and Chemical Engineering, School of Chemistry and Biological Engineering, University of Science and Technology Beijing, Beijing 100083, China; Beijing Key Laboratory for Science and Application of Functional Molecular and Crystalline Materials, Department of Chemistry and Chemical Engineering, School of Chemistry and Biological Engineering, University of Science and Technology Beijing, Beijing 100083, China; School of Materials Science and Engineering, China University of Petroleum (East China), Qingdao 266580, China; School of Materials Science and Engineering, China University of Petroleum (East China), Qingdao 266580, China; Center for High Pressure Science and Technology Advanced Research, Beijing 100193, China; Center for High Pressure Science and Technology Advanced Research, Beijing 100193, China; Beijing Key Laboratory for Science and Application of Functional Molecular and Crystalline Materials, Department of Chemistry and Chemical Engineering, School of Chemistry and Biological Engineering, University of Science and Technology Beijing, Beijing 100083, China

**Keywords:** covalent organic frameworks, robust connection, **tbo** topology, photocatalysis, trapping effect

## Abstract

Constructing 3D functional covalent organic frameworks (COFs) with both robust linkage and planar macrocycle building blocks still remains a challenge due to the difficulty in adjusting both the crystallinity and the dominant 2D structures. In addition, it is also challenging to selectively convert inert C(sp^3^)–H bonds into value-added chemicals. Herein, robust 3D COFs, USTB-28–M (M=Co, Ni, Cu), have been polymerized from the nucleophilic aromatic substitution reaction of *D*_3h_-symmetric 2,3,6,7,14,15-hexahydroxyltriptycene with *D*_4h_-symmetric hexadecafluorophthalocyanine (MPcF_16_) under solvothermal conditions. These chemically stable dioxin-linked COFs show isostructural **tbo** topology made up of three kinds of polyhedron subunits, exhibiting high Brunauer−Emmett−Teller surface areas of ≤1477 m^2^ g^−1^. In particular, the multiple polyhedron subunits in USTB-28–M could trap *N*-hydroxyphthalimide at their corners for easily forming stable phthalimide-*N*-oxyl radicals under visible-light irradiation. The generated radicals efficiently promote the aerobic oxidation of alkyl benzenes with an inert C(sp^3^)–H bond into various ketones. Among the three investigated COFs, the USTB-28–Co radical initiator exhibits the best photocatalytic oxidation activity, converting ethylbenzene into acetophenone with a turnover frequency of 63 h^−1^, which is much higher than those of the monomer CoPcF_16_ (8 h^−1^) and 2D dioxin-linked counterparts (13 h^−1^). This is due to the much prolonged lifetime of the excited state for USTB-28–Co based on the femtosecond transient absorption result. The present work not only presents 3D functional COFs with robust connection and permanent porosity, but also illustrates the uniqueness of porous structures of 3D COFs for high-performance photocatalysis.

## Teaser

This study synthesized robust 3D COFs (USTB-28–Co) through nucleophilic aromatic substitution, achieving high surface areas and efficient photocatalytic oxidation of inert C(sp3)-H bonds under visible light.

## INTRODUCTION

**Scheme 1.**
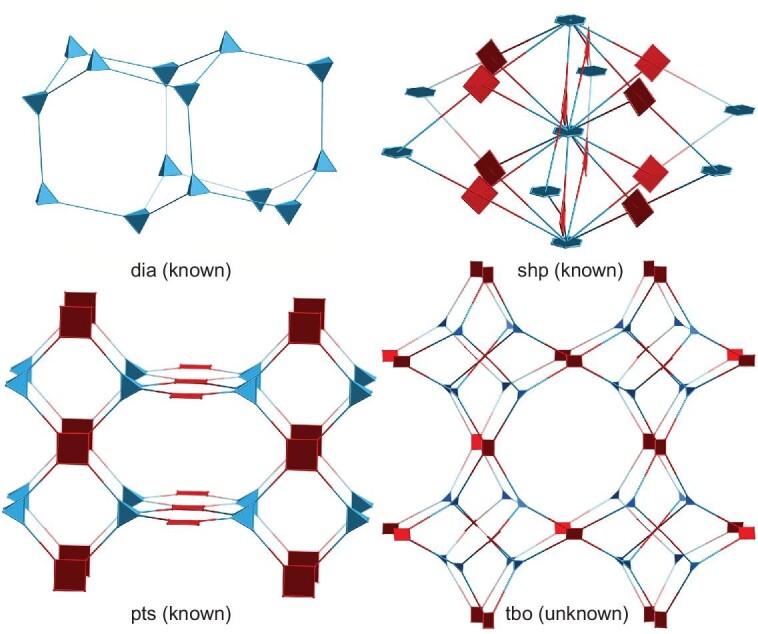
Topology of 3D COFs with robust linkage deduced from direct preparation.

Covalent organic frameworks (COFs) have been emerging as special porous organic polymers with well-defined 2D and 3D crystalline structures since 2005 [1–3]. They show a rapid growth that originates from their immense design space at the atom-precise level and diverse functions such as small molecule adsorption [4,5], heterogeneous catalysis [6,7], optoelectronics [8,9], sensing [10,11] and energy storage [12,13]. During the past nearly two decades, the topological diagram has become an important blueprint for generating COFs with functional building blocks that are controllably located at the knots and edges through dynamic covalent chemistry [14]. The corresponding research attention has gradually moved from new framework design to useful function exploration. However, the chemical robustness of COFs to tolerate harsh conditions is still the hindrance and essential requirement for their development and applications. Dynamic covalent bonds such as boroxine, imine and hydrozone that are derived from reversible reactions are usually not strong enough to survive in high-acid or -base concentrations for maintaining their performance reliability and recoverability. As a consequence, the interest in fabricating stable COFs has inspired researchers to convert these reversible covalent bonds into much stronger connections such as amide [15], imidazole [16,17] and heterocycles [18,19] via post-synthetic modification. In addition, it is also highly desired to develop direct synthesis of stable COFs with irreversible linkages. In this regard, fabrication of crystalline COFs that are derived from irreversible reactions is not easy due to the poor reversibility of these systems, which usually leads to amorphous polymers. Thus far, only a few robust 2D systems have been built by using triazine [20], imide [21‒23], pyrazine [24,25], piperazine [26,27], C=C [6,28,29], phenyl [30] and 1,4-dioxin linkages [31‒33]. This is determined by the few available irreversible reactions for crystallizing COFs and the geometry compatibility between the building blocks. In contrast to 2D counterparts, 3D functional COFs with irreversible connections are much less reported, with, to the best of our knowledge, only six examples that include imide-bonded COFs MPc-PI-COF-3 (M=Co, H_2_) [21], MPc-THHI-COFs (M=H_2_, Ni) [22], PI-COF4 [23] and PI-COF5, imidazole-linked BUCTCOF-7 [17] and sp^2^ carbon-linked BUCT-COF-4 [25], which are based mainly on tetrahedral building blocks (Scheme 1). To enrich the reticular chemistry, it is of significance to develop new approaches for the construction of 3D COFs with irreversible bonds.

The high-performance conversion of inert C(sp^3^)−H bonds in hydrocarbons into value-added chemicals (such as aldehydes or ketones) is of great significance in the field of organic synthesis and the chemical industry [34]. In contrast to traditional methods that use high-energy and corrosive oxidants, the heterogeneous photocatalytic oxidation of C(sp^3^)−H bonds with obtained reactive oxygen species is one of the low-energy and sustainable alternatives due to the use of oxygen and solar light. The carbon radical intermediates that are deduced from C(sp^3^)−H bonds are also able to easily react with unreactive molecular oxygen [35]. Among various approaches, nitroxyl radicals with the help of radical initiators have achieved the high-performance photocatalysis of C−H bonds in hydrocarbons. Exploration of new nitroxyl radical initiators is thus of importance. In recent years, 2D and 3D COFs have been demonstrated to act as new kinds of heterogeneous photocatalysts and photosensitizers due to their semiconducting frameworks, adjustable light absorption scope and well-defined pore structures [36‒39]. In comparison with 2D COFs with 1D channels and packing structures, 3D species with spatially extended networks possess higher specific surface areas, intercrossing pores and more uncovered functional units, which are more helpful in photocatalysis. In addition, the abundant pore corners in 3D COFs may serve as trapping sites during photocatalysis.

On the basis of above considerations, a triptycene building block with a triangular prism shape has been used as a three-connected node to react with four-connected planer macrocycles through irreversible reactions to form robust 3D COFs as radical initiators. Namely, dioxin-linkage USTB-28–M [M=Co(II), Ni(II), Cu(II)] have been prepared through the nucleophilic aromatic substitution of metallic hexadecafluorophthalocyanine (MPcF_16_) and 2,3,6,7,14,15-hexahydroxyltriptycene (HHTC) under solvothermal conditions. This series of isostructural COFs exhibit dioxin-linked **tbo** topological covalent networks, which are assembled from each tritopic HHTC node linking three discrete square MPcF_16_ moieties according to detailed structural investigations. As expected, the high Brunauer–Emmett–Teller (BET) surface areas for these COFs have been determined to be ≤1477 m^2^ g^−1^. Their high surface areas, in combination with the large-sized tetrahedron, truncated cube and truncated cuboctahedron subunits in the **tbo** topological COFs, are favorable for the exposure of more active phthalocyanine sites and diffusion of reagents during catalysis. Interestingly, the **tbo** topological pore structures enable them to adsorb the *N*-hydroxyphthalimide (NHPI) at the pore corners of 3D COFs. Visible-light irradiation of these systems generates the phthalimide-*N*-oxyl (PINO) radical, enabling the aerobic oxidation of alkyl benzenes towards the preparation of valuable ketones with ≤97% yield and ≤99% selectivity. The highly porous 3D **tbo** frameworks as radical initiators and cocatalysts ensure that the COF and PINO synergistically activate and transform the inert C−H bond under visible irradiation and O_2_ atmosphere. The present results not only show the first robust COFs with **tbo** topology and permanent porosities, but also set a synergistic photocatalysis of COFs and nitroxides for challenging the organic transformation of the C−H bond.

## RESULTS AND DISCUSSION

### Design and preparation of USTB-28–M

**Scheme 2.**
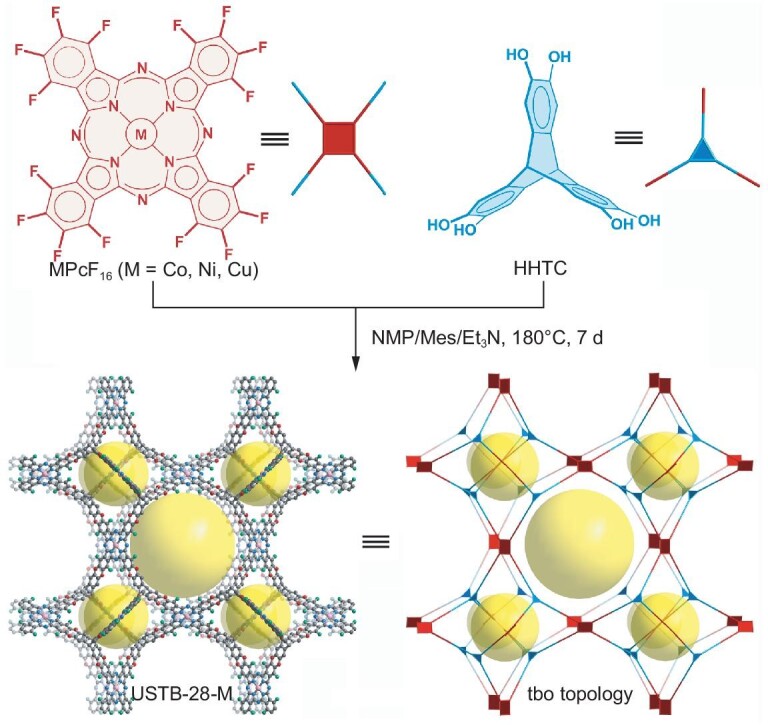
Synthesis and structure of USTB-28–M with **tbo** topology made up of metallophthalocyanine and triptycene building blocks.

**Figure 1.**
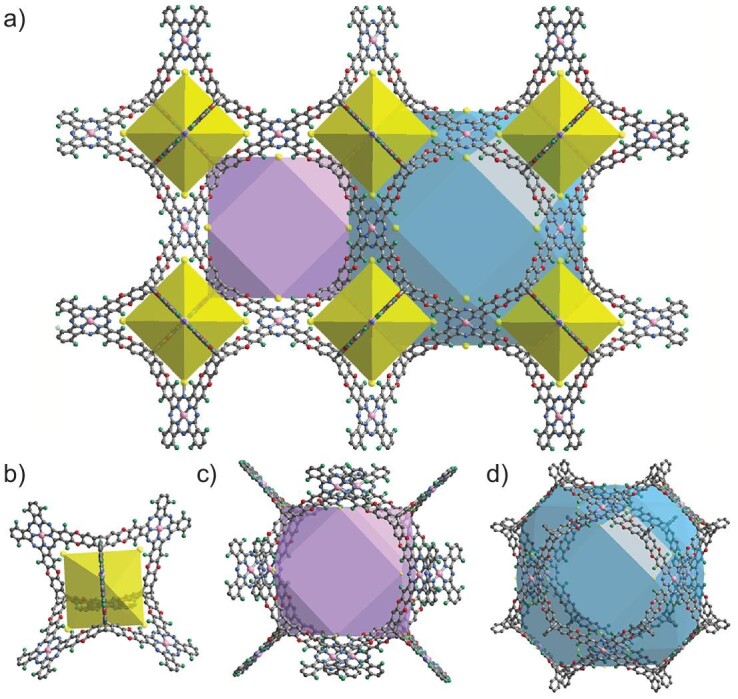
Crystal structure of USTB-28–M (a) showing the polyhedral cages and three types of polyhedral cages (b–d) in USTB-28–M: truncated tetrahedron (yellow), cube (purple) and cuboctahedron (blue).

Phthalocyanines (Pcs) are 18-electron aromatic macrocycles with multifunctionalities that cover from biological, chemical and physical to catalytic areas [32,40‒45]. Their planar *C*_4_ symmetric geometry and eight reactive sites restrain compatible building blocks as well as compatible connection modes, leading to a large fraction of 2D Pc-based COFs and only three 3D frameworks reported thus far [21,22,43]. In the present case, with the inspiration of only one 3D porphyrin **tbo** topology framework [36], the nucleophilic substitution of four-connected planar MPcF_16_ with three-connected triangular prism-shaped HHTC was employed to prepare phthalocyanine **tbo** topology networks with irreversible dioxin-linkage (Scheme 2 and Fig. 1). In addition, the multifunctionalities of phthalocyanines enable these 3D COFs to be used as sensitizers and/or catalysts for photocatalysis. However, it should be noted that the preparation of crystalline samples with irreversible connections is a difficult task. After a variety of reaction conditions were screened, black powders of USTB-28–M were successfully obtained through the [3 + 4] nucleophilic substitution polymerization of MPcF_16_ with HHTC in a mixed solution of *N*-methyl-2-pyrrolidone/mesitylene/triethylamine (NMP/Mes/Et_3_N) at 180°C for 7 days (Fig. S1 and Table S1). Notably, the high temperature and optimal organic solvent proportion are critical for the generation of high-crystallinity COFs. These three COFs possess good solvent resistance in water, HCl, NaOH and common organic solvents such as NMP, dichloromethane (DCM), ethanol, tetrahydrofuran and dimethylformamide (Figs S2–S4). Additionally, these materials have good thermal stability, as indicated by the thermogravimetric analytic curves with decomposing temperature at ∼350°C (Figs S5–S7).

### Crystal structure and permanent porosity

**Figure 2.**
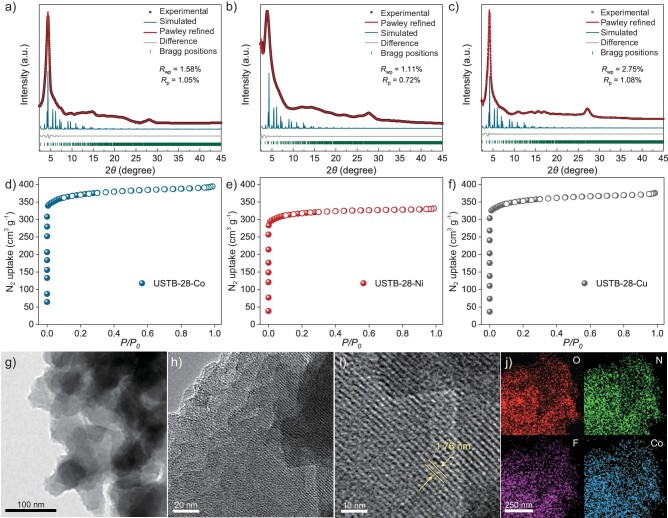
(a)–(c) PXRD patterns of USTB-28–Co, USTB-28–Ni and USTB-28–Cu [experimental PXRD profile (black), refined profile (red), simulation pattern (blue), the difference between the experimental and refined PXRD (gray) and the Bragg positions (green)], respectively. (d–f) N_2_ adsorption (solid) and desorption (hollow) curves of USTB-28–M (M=Co, Ni, Cu) at 77 K. (g) TEM images of USTB-28–Co. (h) and (i) HRTEM images of USTB-28–Co. (j) EDX mapping images of USTB-28–Co.

On the basis of the geometries of the precursors and the connection, the **tbo** topological structure models of COFs were built by using the Materials Studio software package (Scheme 2 and Fig. 1). USTB-28–Co has a cubic *Fm*–3*m* space group. Four-connected square MPcF_16_ and three-connected trigonal HHTC building units are connected through dioxin linkages to form tetrahedron subunits as well as truncated cube and cuboctahedron subunits, thus extending into a 3D framework with the **tbo** topology. The periodical structures of three 3D COFs were determined by using powder X-ray diffraction (PXRD) measurements in conjunction with structural simulations (Fig. 2a‒c). USTB-28–Co exhibits an intense peak at 4.14° and a weak peak at 27.61° (Fig. 2a), revealing its crystalline nature with the ordered arrangement of building blocks after polymerization. Similar PXRD patterns were determined for USTB-28–Ni and USTB-28–Cu (Fig. 2b and c). The experimental PXRD pattern is consistent with the simulated one on the basis of the non-interpenetrated model rather than the 2-fold interpenetration frameworks (Figs S8 and S9). Notably, the size of the cavities can only allow the 2-fold interpenetration of the Pc-based **tbo** framework, denying the occurrence of multiple interpenetrations. The biggest window size of **tbo** topological USTB-28–M is 13.3 and 23.5 Å, respectively, which is favorable for reagent diffusion during catalysis. A Pawley refinement upon the PXRD data of USTB-28–Co shows unit cell parameters of *a* = *b* = *c* = 70.3 Å, *α* = *β* = *γ* = 90° with *R*_wp_ = 1.58% and *R*_p_ = 1.05%, illustrating the satisfactorily low residual values and acceptable profile differences. This is also true for USTB-28–Ni (*a* = *b* = *c* = 70.4 Å, *α* = *β* = *γ* = 90°, *R*_wp_ = 1.11% and *R*_p_ = 0.72%) and USTB-28–Cu (*a* = *b* = *c* = 70.4 Å, *α* = *β* = *γ* = 90°, *R*_wp_ = 2.75% and *R*_p_ = 1.08%). USTB-28–M actually represent the first examples of phthalocyanine-based COFs with **tbo** topology and the second COFs with **tbo** topology, enriching the structural diversity. Prior to the measurement of gas sorption isotherms, USTB-28–M were degassed under a high vacuum to eliminate the guest molecules in the pores. These compounds exhibit type-I sorption curves (Fig. 2d‒f). The BET and Langmuir surface areas of activated USTB-28–Co were found to be 1477 and 1705 m^2^ g^−1^, respectively. Slightly lower BET and Langmuir surface areas were revealed for USTB-28–Ni, at 1270 and 1438 m^2^ g^−1^, and for USTB-28–Cu, at 1399 and 1623 m^2^ g^−1^. The pore size distribution of USTB-28–Co has been fitted on the basis of a nitrogen adsorption curve at 77 K, showing three kinds of pores with sizes of 1.90, 2.84 and 3.80 nm, respectively. These data are consistent overall with the three theoretical void sizes of ∼1.60, ∼2.80 and ∼3.60 nm (Fig. S10). This is also true for USTB-28–Ni and USTB-28–Cu. These results further support the above-proposed **tbo** COF models. However, their much smaller experimental pore volumes (0.61 cm^3^ g^−1^) than the theoretical values are possibly due to the presence of amorphous oligomers in the pores.

### Spectroscopic and morphology characterization of USTB-28–M

Fourier transform infrared (FTIR) spectra of USTB-28–M have been collected (Figs S11‒S13). In comparison with that of monomers, the FTIR spectrum of USTB-28–Co shows new absorption bands at 1258 and 1054 cm^−1^ due to the dioxin C−O asymmetric and symmetric stretching modes [31,32], respectively. Similarly, the characteristic stretching vibration bands were observed at 1261 and 1050 cm^−1^ for USTB-28–Ni and 1259 and 1058 cm^−1^ for USTB-28–Cu. The formation of the dioxin linkages in the 3D COF was further checked by applying solid-state ^19^F magic-angle-spinning nuclear magnetic resonance spectroscopy (^19^F MAS NMR) to the USTB-28–Ni sample. The ^19^F MAS NMR spectrum of USTB-28–Ni displays only one distinct peak at −126.87 ppm (Fig. S14), which is completely different from the precursor of phthalocyanine (namely tetrafluorophthalonitrile) with two kinds of fluorine atoms [32], further indicating the successful connection between these two kinds of building blocks. Scanning electron microscopy (SEM) images determine the morphologies of uniform aggregated particles with sizes of 0.5–2.0 μm for these three COFs (Fig. S15). Transmission electron microscopy (TEM) was also used to characterize the morphology of USTB-28–M COFs, which showed microcrystalline particles (Fig. 2g and Fig. S16). The crystalline character of USTB-28–Co was visualized on the basis of the lattice fringe that was collected through high-resolution transmission electron microscopy (HRTEM). As shown in Fig. 2h and i, USTB-28–Co exhibits clear lattice fringes of 1.76 nm due to the (400)/(040)/(004) diffraction. A similar lattice fringe of 1.78 nm was determined for USTB-28–Ni and USTB-28–Cu. Energy-dispersive X-ray spectroscopy (EDX) analysis discloses that the O, N, F and Co atoms uniformly cover the USTB-28–Co sample (Fig. 2j). The nickel and copper elements in a uniform distribution mode are also spread over USTB-28–Ni and USTB-28–Cu samples in EDX images (Fig. S16).

The X-ray photoelectron spectroscopy (XPS) analysis estimates the surface chemical states and elemental compositions of these USTB-28–M COFs (Figs S17‒S20). The O, N, F and corresponding metallic elements emerge in the full XPS spectra of USTB-28–M (M=Co, Ni, Cu). For the high-resolution Co 2p XPS spectrum of USTB-28–Co, the binding energies at 780.5 and 796.1 eV are due to two distinctive Co 2p_3/2_ and Co 2p_1/2_ peaks for the divalent metal. As expected, the binding energies of the divalent nickel and copper elements occur at 855.3 and 873.4 eV, and 934.9 and 954.7 eV, respectively, corresponding to their 2p_3/2_ and 2p_1/2_ core levels [32]. To further disclose the valence states and coordination environments of the Co, Ni and Cu atoms in these COFs, X-ray absorption spectroscopy and extended X-ray absorption fine structure (EXAFS) analyses were conducted. As shown in Fig. S21a, the Co K-edge X-ray absorption near-edge structure (XANES) spectrum of USTB-28–Co gives a typical Co(II) peak at ∼7725 eV, similarly to the commercial divalent cobalt phthalocyanine compound. This implies the presence of a planar four-coordination environment around the cobalt ion and its divalent oxidation state. Moreover, the EXAFS spectrum of USTB-28–Co (Fig. S21b) displays an intensive peak at ∼1.35 Å, demonstrating the presence of complexation between the Co and N atoms in this COF. On the basis of a planar square Co–N_4_ coordination model, the fitting gives the average distance of Co–N as 1.99 Å, suggesting the maintenance of the metallophthalocyanine segment in this COF. Similar phenomena are found for the Ni and Cu K-edge XANES spectra of USTB-28–M (M=Ni, Cu) (Figs S22 and S23), showing the typical divalent Ni and Cu peaks at 8336 and 8982 eV (Table S2), respectively. Inductively coupled plasma spectrometry analysis determines the Co, Ni and Cu contents in USTB-28–M at values of 4.91, 4.92 and 5.24 wt % (as listed in Table S3), corresponding very well to the theoretical values of 5.05, 5.03 and 5.43 wt %, respectively. In addition, the elemental analyses show similar experimental and theoretical data of N elements for these three samples (Table S4), confirming the generation of the designed **tbo** topological COFs based on reticular chemistry.

### Photocatalytic performance of USTB-28–M

Phthalocyanine and its metallic derivatives represent excellent visible-light photosensitizers, having remarkable activity to bind and activate molecular oxygen [35,46]. In the present case, USTB-28–M were employed as radical initiators to convert NHPI into PINO for promoting the aerobic oxidation of alkyl benzenes. The heterogeneous photocatalysis of USTB-28–M was performed in acetonitrile solution containing NHPI under O_2_ atmosphere and visible-light irradiation. As shown in Table 1, a combination of USTB-28–Co (0.002 mmol) and NHPI (0.02 mmol) can promote the ethylbenzene (EB) oxidation to form acetophenone in a yield of 59% after 2.0 h (Table 1, entry 1). As shown in Table S5, another three solvents, namely methanol, ethanol and ethyl acetate, were screened for comparison and exhibited much lower yields of 39%, 22% and 15%, respectively. In acetonitrile solution, diminishment of either the USTB-28–Co (0.001 mmol) or the NHPI (0.01 mmol) amount induces a serious decrease in the product yields to 12% and 41% (Table 1, entries 2 and 3), respectively. In contrast, extension of the reaction time to 4.0 and 8.0 h leads to enhanced acetophenone yields of 79% and 97% (Table 1, entries 4 and 5), respectively. The high selectivity of 99% for the conversion to form acetophenone was determined.

**Table 1.** Photocatalysis towards the aerobic oxidation of ethylbenzene with various catalysts under different conditions.^a,b^

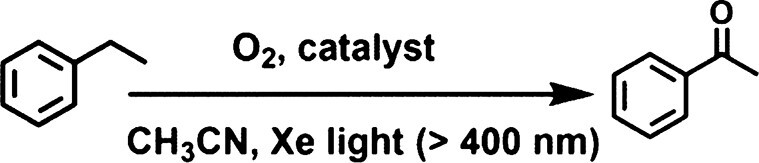			
		Time	Yield
Entry	Catalyst	(h)	(%)
1	USTB-28–Co + NHPI	2.0	59
2^c^	USTB-28–Co + NHPI	2.0	12
3^d^	USTB-28–Co + NHPI	2.0	41
4	USTB-28–Co + NHPI	4.0	79
5	USTB-28–Co + NHPI	8.0	97
6^e^	USTB-28–Co + NHPI	2.0	–
7^f^	USTB-28–Co + NHPI	2.0	–
8^g^	USTB-28–Co + NHPI	8.0	–
9	USTB-28–Co	2.0	–
10	NHPI	2.0	–
11	USTB-28–Ni + NHPI	8.0	61
12	USTB-28–Cu + NHPI	8.0	62
13	CoPcF_16_ + NHPI	8.0	27
14	CoPc–O–COF + NHPI	8.0	34
15^h^	USTB-28–Co + NHPI	2.0	53
16^i^	USTB-28–Co + NHPI	2.0	51
17^j^	USTB-28–Co + NHPI	2.0	–

a Reaction conditions: substrate (0.3 mmol), USTB-28–M (0.002 mmol based on metallophthalocyanine unit) and *N*-hydroxyphthalimide (0.02 mmol), acetonitrile (2.0 mL), O_2_ (1 atm), irradiated by a 350-W Xe lamp. ^b^The yields of products were determined by gas chromatography (GC) areas with external standards; all results are yields. ^c^USTB-28–Co (0.001 mmol based on cobalt phthalocyanine unit). ^d^*N*-hydroxyphthalimide (0.01 mmol). ^e^In dark. ^f^In N_2_. ^g^The filtrate of USTB-28–Co + NHPI. ^h^In the presence of AgNO_3_. ^i^In the presence of BQ. ^j^In the presence of NaHCO_3_.

**Figure 3.**
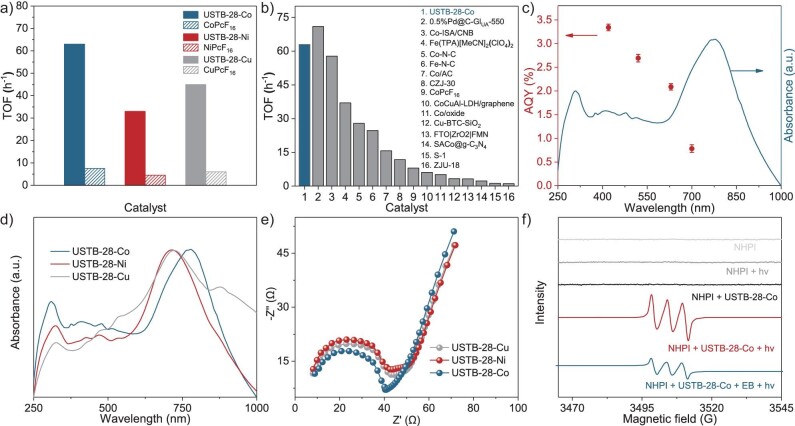
(a) TOFs for MPcF16 and USTB-28–M (M=Co, Ni, Cu) calculated in the first 1.0 h of photocatalysis. (b) Comparison of catalytic performances in various catalysts. Their corresponding references are given in Table S8. (c) Wavelength-dependent apparent quantum yield (AQY) of photocatalytic oxidation of aryl alkanes on USTB-28–Co. (d) UV−vis diffuse reflectance spectroscopy (DRS) absorption spectra of USTB-28–Co, USTB-28–Ni and USTB-28–Cu. (e) Nyquist plots of USTB-28–Co, USTB-28–Ni and USTB-28–Cu. (f) EPR data under the irradiation of a 350-W Xe lamp.

In the control experiments, the absence of O_2_ and light or the utilization of only USTB-28–Co or only NHPI results in the detection of no product (Table 1, entries 6–10), implying the important role of the present reaction conditions, in particular the synergistic photocatalysis system. In addition, EB is unable to be converted upon the filtrate of an acetonitrile suspension of USTB-28–Co and NHPI for 8 h under the same conditions, disclosing the heterogeneous nature. Replacement of USTB-28–Co with USTB-28–Ni and USTB-28–Cu induces a decreased yield of acetophenone to 61%–62% under the same conditions (Table 1, entries 11 and 12) due to the better activity of the metallic phthalocyanine units in the former COF than the latter two species towards oxygen activation. Furthermore, in contrast to USTB-28–Co, monomeric CoPcF_16_ and a 2D dioxin-linked phthalocyanine COF, CoPc–O–COF, as shown in Scheme S2 and Fig. S24, have worse photocatalytic behaviors in terms of lower yields of 27% and 34% (Table 1, entries 13 and 14), respectively. Especially, the turnover frequency (TOF) of USTB-28–Co (63 h^−1^) is much higher than that for monomeric CoPcF_16_ (8 h^−1^) and CoPc–O–COF (13 h^−1^) during the first 1 h of photocatalysis due possibly to the highly porous structure and well-dispersed non-aggregated phthalocyanine subunits that originated from the **tbo** COF (Fig. 3a and Table S6). Similar phenomena, namely higher TOF values for COFs, are also observed for USTB-28–Ni and USTB-28–Cu in comparison with those for their monomers. Herein, it is worth noting that the present synergistic photocatalysis system that is made up of USTB-28–Co and NHPI shows prominent photocatalytic activity in terms of a TOF value of 63 h^−1^, surpassing various reported photocatalysis and thermocatalysis systems, including Co-ISA/CNB (58 h^−1^) [47], Fe(TPA)[MeCN]_2_(ClO_4_)_2_ (37 h^−1^) [48] and Co-N-C (28 h^−1^) [49], and comparable to 0.5%Pd@C-Glu_A_-550 (71 h^−1^) [50] (Fig. 3b).

The wavelength-dependent apparent quantum yield of acetophenone production upon USTB-28–Co is located in the range of 0.8%–3.3% (Fig. 3c). USTB-28–Co could successively promote six runs of EB oxidation, maintaining high photocatalytic efficiency in a yield of >93% (Table S7). In contrast to the as-prepared USTB-28–M, the stability of the used COFs after photocatalysis was supported by PXRD data, FTIR spectra and TEM images (Figs S25‒S30). In order to understand the reaction mechanism, the controls were conducted in the presence of electron (e^−^), radical and hole (h^+^) scavengers, respectively. The introduction of an e^−^ capture reagent (AgNO_3_) and O_2_^•−^ quencher [1,4-benzoquinone (BQ)], respectively, into EB oxidation upon USTB-28–Co and NHPI results in a slightly decreased yield of acetophenone from 59% to 51%–53% after 2 h under the same conditions. These experimental results preclude the decisive role of the photogenerated electron and O_2_^•−^ radical in the present photocatalytic system. In contrast, the addition of an h^+^ capture reagent (NaHCO_3_) seriously suppresses the production of acetophenone, illustrating the important role of photogenerated h^+^ in the oxidation reaction to generate the radical [35].

Furthermore, the oxidation of various aryl alkanes, including another 10 substrates to corresponding ketones, was checked by using these COFs under the optimal conditions (as listed in Table 2). In contrast to EB, the introduction of substituents seems to make the oxidation efficiency slightly worse after 8.0 h for five derivatives, including propyl benzene, 4-methoxyethylbenzene, 3-bromoethylbenzene, 4-nitroethylbenzene and 4-ethyl-1,1'-biphenyl, with product yields of 71%–97% (Table 2, entries 1–6). Obviously, the electron-withdrawing nitro group seriously decreases the oxidation product yield to 71%. Tetralin, indane and 9*H*-fluorene are oxidized into corresponding ketones in yields of 84%–95% due possibly to their different dissociation energies of hydrogen atoms (Table 2, entries 7–9). Due to the steric hindrance, the product yield (87%) of diphenylmethane oxidation is lower than that of 9*H*-fluorene (95%) (Table 2, entries 9 and 10). This point is confirmed by the detected higher yield of 96% for 9*H*-xanthene oxidation (Table 2, entry 11).

**Table 2.** Photocatalytic oxidation of benzylic sp^3^ C–H bonds of aryl alkanes.^a,b^

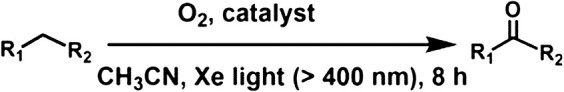		
Entry	Product	Yield (selectivity)^c^
1	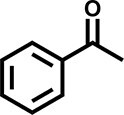	97 (99)/61 (76)/62 (70)
2	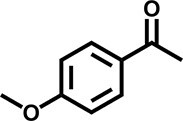	88 (95)/31 (61)/36 (60)
3	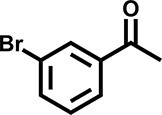	78 (98)/31 (69)/38 (75)
4	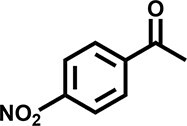	71 (87)/33 (67)/37 (77)
5	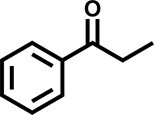	81 (97)/31 (68)/49 (69)
6	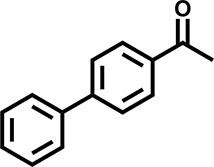	94 (94)/34 (70)/59 (80)
7	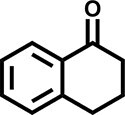	94 (99)/44 (64)/49 (80)
8	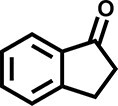	84 (88)/43 (57)/50 (68)
9	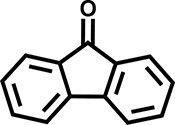	95 (96)/41 (67)/41 (65)
10	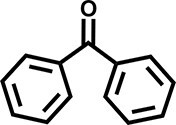	87 (90)/24 (61)/34 (62)
11	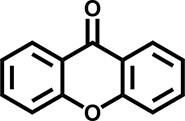	96 (98)/30 (67)/41 (71)

a Reaction conditions: substrate (0.3 mmol), USTB-28–M (0.002 mmol based on monomeric metallophthalocyanine unit), *N*-hydroxyphthalimide (0.02 mmol), acetonitrile (2.0 mL), O_2_ (1 atm), irradiated by a 350-W Xe lamp for 8 h. ^b^The yields of products were determined by GC areas with external standards; all results are yields. ^c^Yield for USTB-28–M (M=Co, Ni, Cu), respectively.

### Photocatalytic mechanism

The present work establishes a high-performance synergistic photocatalytic system towards the oxidation of aryl alkanes with an inert C–H bond. According to previous investigation results [35], NHPI tends to generate an intramolecular hydrogen-bonding interaction with its one carbonyl group and its high R_2_NO–H bond dissociation enthalpy of ∼368.4 kJ·mol^−1^ (∼3.8 eV in CH_3_CN) is too high for direct homolysis by ˃400 nm of irradiation (< 3.1 eV). The adsorption of NHPI on the pore surface of COFs may achieve oxidation by holes to afford the PINO radical. In contrast to free NHPI, the NHPI that is adsorbed onto the USTB-28–Co pore surface was detected by the observation of a C=O vibration shift from 1705 to 1726 cm^−1^. The apparent red shift of O−H vibration from 3138 to 3035 cm^−1^ suggests the activation of O−H in NHPI by the USTB-28–Co (Fig. S31). In contrast to USTB-28–Co, the apparent blue shift of the dioxin C−O asymmetric and symmetric stretching vibration from 1258 and 1054 cm^−1^ to 1267 and 1063 cm^−1^ for the NHPI-adsorbed USTB-28–Co sample implies the presence of an interaction between these oxygen atoms and the NHPI molecule in the latter system (Fig. S32). This is also true for the NHPI-adsorbed USTB-28–Ni and USTB-28–Cu (Figs S33‒S36). This point is also consistent with the theoretical result that shows multiple interactions between NHPI and a COF fragment made up of 1,4,8,11,15,18,22,25-octafluorophthalocyaninato cobalt substituted by one 2,3-dihydroxyltriptycene. It is worth noting that H bond (2.109 Å), π–π (3.103 Å) and C–H···π (2.394 Å) interactions exist between the USTB-28–Co fragment and NHPI. The moderately strong interaction between these two molecules is supported by an adsorption energy of −1.0 eV, which is responsible for trapping NHPI molecules in the **tbo** COFs (Figs S37 and S38). In good contrast, there is no trapped NHPI molecule in the solid monomer CoPcF_16_ and 2D CoPc–O–COF according to their IR data (Figs S39–S41). As a result, USTB-28–Co with trapped NHPI enables better photocatalytic activity than that of the monomer and 2D COF counterpart. Nevertheless, the highly porous structure of 3D COFs is also helpful for reagent diffusion and, in turn, photocatalysis.

It is certainly of significance to explore the intrinsic electronic structures of COFs. Firstly, diffuse reflectance Ultraviolet-visible (UV-vis) spectra of these COFs and phthalocyanine monomers were collected and are shown in Fig. 3d and Fig. S42. CoPcF_16_ has strong absorption bands at 310 and 665 nm due to the typical S and Q bands for phthalocyanine compounds. After polymerization with the HHTC building blocks, a wide absorption band with a maximum peak at 779 nm is observed for USTB-28–Co and the Q-band tail of the phthalocyanine ends at 1000 nm. The bathochromic shift of the Q band indicates the generation of an electronic conjugated framework. Similar wide absorption bands are detected for USTB-28–Ni and USTB-28–Cu. On the basis of the Kubelka–Munk formula, the band gaps of these three COFs were calculated to be ∼2.16, ∼2.08 and ∼2.06 eV, respectively, indicating their good photocatalytic nature due to their wide light absorption scope (Figs S43‒S45). The Mott−Schottky (MS) curves of USTB-28–M were fitted to acquire their conduction bands at the positions of −0.32, −0.31 and −0.31 eV vs. the normal hydrogen electrode (NHE) for USTB-28–Co, USTB-28–Ni and USTB-28–Cu (Figs S46‒S48), respectively. With the help of the above optical gaps for these three COFs, the valence bands were therefore predicted to be 1.54, 1.47 and 1.45 V (Fig. S49), ensuring the photo-induced electron transfer for COFs to obtain the reactive radical PINO (*E*_ox_ = 1.10 V vs NHE) [46].

The photocurrent response measurements were carried out to investigate the charge separation efficiency of as-prepared COFs. As shown in Fig. S50, USTB-28–Co exhibits a stronger photocurrent than USTB-28–Ni and USTB-28–Cu under the same operation conditions, revealing enhanced electron−hole separation. This point is also supported by the fact that USTB-28–Co shows a semicircle with a smaller radius than those of USTB-28–Ni and USTB-28–Cu according to the electrochemical impedance spectroscopy measurements (Fig. 3e). Among the three COFs, the highest formation efficiency of holes for USTB-28–Co is beneficial to its prominent photocatalytic activity for cooperative photocatalysis with the *N*-oxyl radical.

Subsequently, the electron paramagnetic resonance (EPR) spectra of the acetonitrile solutions/suspensions that contain various reagents were determined at room temperature in air. Either in the presence or absence of irradiation, there is no signal for the acetonitrile of NHPI, excluding the production of PINO. After the addition of USTB-28–Co into the above solutions, there is still no EPR signal in the lack of irradiation. Irradiation breaks the EPR silence to afford a triplet hyperfine splitting signal (Fig. 3f). The *g*-factor of this triplet signal is 2.0085, indicating the formation of PINO radical (*g* = 2.0073). The introduction of EB significantly reduces the intensity of the signal, hinting that the reaction has proceeded. When the EPR test of NHPI and USTB-28–Co was carried out in N_2_, the observed triplet EPR signals confirmed the generation of PINO radical (Fig. S51). This is consistent with the investigation result reported previously [35], showing that the photo-driven conversion from NHPI into PINO does not rely on oxygen, supporting the capability of COFs in forming PINO. This is also true for USTB-28–Ni and USTB-28–Cu but with relatively weak PINO signals under the same conditions. These results indicate that USTB-28–Co exhibits stronger ability than USTB-28–Ni and USTB-28–Cu to oxidize NHPI into PINO radical. To verify the formation of O_2_^•−^, a trapping experiment that contained 5,5-dimethyl-1-pyrroline *N*-oxide (DMPO) in the acetonitrile suspensions of USTB-28–M determines the typical 6-fold characteristic peak for DMPO-O_2_^•−^ under light irradiation (Fig. S52).

**Figure 4.**
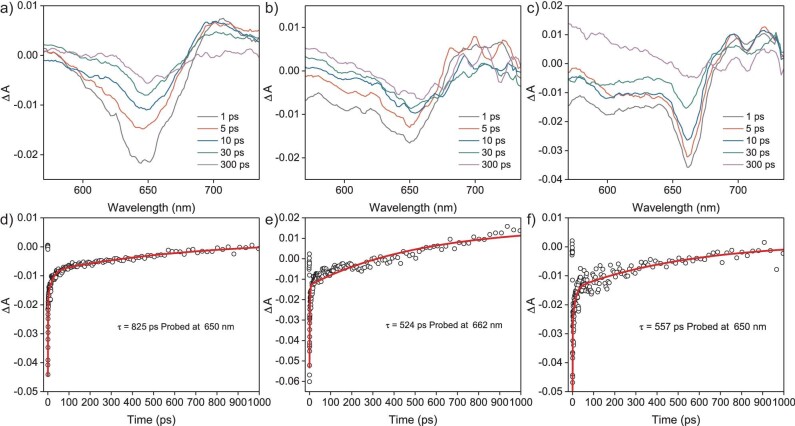
The fs-TA spectra of (a) USTB-28–Co, (b) CoPcF_16_ and (c) CoPc–O–COF in N_2_ atmosphere excited by a 500-nm laser. The kinetic trace of (d) USTB-28–Co, (e) CoPcF_16_ and (f) CoPc–O–COF at 650, 662 and 650 nm, respectively.

Femtosecond transient absorption (fs-TA) spectra tests were performed to investigate the photoexcitation processes and the formation of excited-state species in different catalysts. Under 500 nm of laser excitation, USTB-28–Co exhibits broad excited-state absorption (ESA) bands and ground-state bleaching (GSB) bands at 700 and 650 nm (Fig. 4a), respectively, in a nitrogen atmosphere. In contrast, CoPcF_16_ displays much narrower GSB bands and the intensity of the GSB bands for CoPc–O–COF with a 2D structure was weaker (Fig. 4b and c). Such observations are attributed to the unique 3D conjugated electronic nature of USTB-28–M. The GSB band for USTB-28–Co at 650 nm has a lifetime of 825 ps, corresponding to the singlet state going back to the ground state, which is significantly longer than those of CoPcF_16_ (524 ps) and CoPc–O–COF (557 ps). This is consistent with the kinetic decay process that was probed at 700 nm (Fig. 4d–f and Fig. S53). These results illustrate the formation of 3D COF with an extended excited-state lifetime, which is beneficial for photo-driven charge separation and the formation of more holes for converting NHPI into PINO radical. Additionally, when USTB-28–Co was exposed to an oxygen atmosphere and then NHPI was added, both the ESA and GSB lifetimes were seriously reduced, confirming the disturbance of O_2_ and NHPI in the charge-transfer process of this COF (Figs S54‒S56). This finding is in agreement with the results from the above-mentioned EPR tests.

### Theoretical calculations

**Figure 5.**
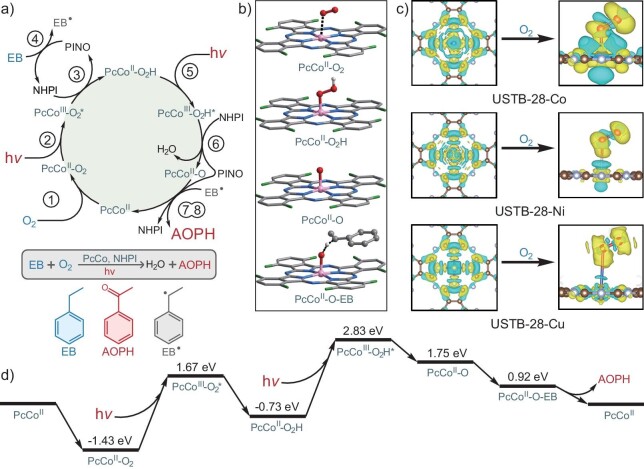
(a) The full mechanism of the photocatalytic EB→AOPH reaction. The numbers in circles represent the reaction equations in the text. For example, ① means Equation (1). (b) Intermediate structures. (c) Differential charge density maps of USTB-28–Co, USTB-28–Ni and USTB-28–Cu, and the charge density differences of adsorbed O_2_ on USTB-28–Co, USTB-28–Ni and USTB-28–Cu, respectively. The yellow and cyan areas represent electron gain and loss. (d) Gibbs free energy of the photocatalytic EB→AOPH reaction.

The π-electron localized orbital locator (π-LOL) calculation data are shown in Fig. S57. Although HHTC possesses a 3D triangular prism structure, the uniform π-electron delocalization covers the whole HHTC skeleton, suggesting the 3D homoaromatic conjugated electronic system behavior of the HHTC molecule. The integration of a 3D conjugated HHTC skeleton with a 2D fully conjugated MPc unit through dioxin-linkage results in USTB-28–M with an enlarged π-conjugated system relative to that of MPcF_16_. The conjugated electronic nature of these 3D COFs is also confirmed by the moderately high conductivities of 4.2 × 10^−5^, 8.7 × 10^−6^ and 7.1 × 10^−6^ S cm^−1^ for USTB-28–Co, USTB-28–Ni and USTB-28–Cu, respectively. These values are comparable to those for conjugated 3D COFs [23‒29,35,46,47], further indicating the π-electron delocalization nature of all the 3D **tbo** topological frameworks. This structural feature is beneficial for the generation of the charge-separated state for COFs and thus the photocatalytic efficiency. In order to further clarify the photocatalytic mechanism, calculations of the Gibbs free energy for each reaction pathway were made. It is worth noting that these COFs are able to generate PINO radical even in the absence of O_2_, as mentioned above. However, the photocatalytic reaction was carried out under an O_2_ atmosphere in the present case. As a result, the role of O_2_ was also considered in the photocatalytic mechanism. As shown in Fig. 5, the full mechanism of the photocatalytic EB → acetophenone (AOPH) process could be divided into three stages: (i) the first step is energy input and transfer, including photon capture and PcCo-O_2_-H generation; (ii) the second step is energy input and transfer for the generation of the oxo–metal intermediate PcCo–O driven by the second photon; and (iii) the third step is energy output due to the formation of AOPH.

First, PcCo^II^–O_2_ formed after PcCo^II^ binding to an oxygen molecule captures a photon in the visible-light range to generate the excited-state PcCo^III^–O_2_*^•−^ with an energy barrier of ∼3.1 eV. Immediately, the NHPI molecule in the acetonitrile approaches the PcCo^III^–O_2_*^•−^ and contributes an H^+^ ion, leading to an unstable intermediate (PcCo^III^–O_2_H)^+^–(PINO^−^). After the rapid electron transfer from PINO^−^ to the Co^III^ site in the metallophthalocyanine unit, a new pair of (PcCo^II^–O_2_H)–(PINO) appears in the reaction system. In the next moment, PINO takes a hydrogen atom from EB→EB^•^, recovering back to NHPI. In a word, the first energy-inputting stage brings the correct energy and provides the first pathway for NHPI oxidation with an energy decrease of 2.40 eV, as shown in Equations (1)–(4):


(1) \begin{eqnarray*}
{\mathrm{PcC}}{{{\mathrm{o}}}^{{\mathrm{II}}}} + {{{\mathrm{O}}}_2} \to {\mathrm{PcC}}{{{\mathrm{o}}}^{{\mathrm{II}}}} -{{{\mathrm{O}}}_2},\end{eqnarray*}

(2) \begin{eqnarray*}
{\mathrm{PcC}}{{{\mathrm{o}}}^{{\mathrm{II}}}} -{{{\mathrm{O}}}_2} + hv \to {\mathrm{PcC}}{{{\mathrm{o}}}^{{\mathrm{III}}}} -{{{\mathrm{O}}}_2}^{* \bullet} { }^ -, \end{eqnarray*}

(3) \begin{eqnarray*}
{\mathrm{PcC}}{{{\mathrm{o}}}^{{\mathrm{III}}}} -{{{\mathrm{O}}}_2}^ {* \bullet}{ }^ - + {\mathrm{NHPI}} \to {\mathrm{PcC}}{{{\mathrm{o}}}^{{\mathrm{II}}}} -{{{\mathrm{O}}}_2}{\mathrm{H}} + {\mathrm{PINO}},\\
\end{eqnarray*}

(4) \begin{eqnarray*}
{\mathrm{EB}} + {\mathrm{PINO}} \to {\mathrm{E}}{{{\mathrm{B}}}^ \bullet } + {\mathrm{NHPI}}.
\end{eqnarray*}

The reaction continues from the exciting process of the new state for photosensitizer PcCo^II^–O_2_H. Driven by the second step of the energy-inputting procedure that is brought about by the second photon-capturing process, the stable intermediate PcCo^II^–O_2_H is reactivated to PcCo^III^–O_2_H*^•^ with an energy of 2.83 eV and continues to oxidize NHPI to PINO. Similarly to the first pathway for the NHPI oxidation process, another NHPI molecule is also oxidized to PINO with an energy decrease of 1.08 eV, as shown in Equations (5) and (6):


(5) \begin{eqnarray*}
{\mathrm{PcC}}{{{\mathrm{o}}}^{{\mathrm{II}}}} -{\mathrm{OOH}} + hv \to {\mathrm{PcC}}{{{\mathrm{o}}}^{{\mathrm{III}}}} -{\mathrm{OOH}}^*, \end{eqnarray*}

(6) \begin{eqnarray*}
{\mathrm{PcC}}{{{\mathrm{o}}}^{{\mathrm{III}}}} -{\mathrm{OOH}}^* + {\mathrm{NHPI}} \to {\mathrm{PcC}}{{{\mathrm{o}}}^{{\mathrm{II}}}} -{\mathrm{O}}\\
\qquad +\, {{{\mathrm{H}}}_2}{\mathrm{O}} + {\mathrm{PINO}}.
\end{eqnarray*}

Until now, the system has prepared the two active moieties EB^•^ and PcCo^II^–O, via Equations (4) and (6), which combine into the final intermediate PcCo^II^–O–EB^•^. In this process, the left hydrogen atom that is located on the secondary carbon atom in the EB^•^ radical is activated. Finally, PINO takes away this hydrogen atom from EB^•^, constructing the final product, AOPH, as show in Equations (7) and (8):


(7) \begin{eqnarray*}
{\mathrm{PcC}}{{{\mathrm{o}}}^{{\mathrm{II}}}} -{\mathrm{O}} + {\mathrm{E}}{{{\mathrm{B}}}^ \bullet } \to {\mathrm{PcC}}{{{\mathrm{o}}}^{{\mathrm{II}}}} -{\mathrm{O}} -{\mathrm{E}}{{{\mathrm{B}}}^ \bullet },\end{eqnarray*}

(8) \begin{eqnarray*}
&&{\mathrm{PcC}}{{{\mathrm{o}}}^{{\mathrm{II}}}} -{\mathrm{O}} -{\mathrm{E}}{{{\mathrm{B}}}^ \bullet } + {\mathrm{PINO}}\\
&&\qquad \to {\mathrm{NHPI}} + {\mathrm{PcC}}{{{\mathrm{o}}}^{{\mathrm{II}}}} + {\mathrm{AOPH}},
\end{eqnarray*}

${{{\mathrm{O}}}_2} + {\mathrm{EB}} \to {{{\mathrm{H}}}_2}{\mathrm{O}} + {\mathrm{AOPH}}$ (Total Eq.).

In the above theoretical analysis, we describe the detailed reaction pathway in EB oxidation based on USTB-28–Co. In order to rationalize the excellent photocatalytic behavior of USTB-28–Co, a comparative investigation of USTB-28–Co/Ni/Cu was also carried out regarding oxygen capture at the same level of M06-2X/6–311 + G(d, p). According to the calculation results, the Co–O bond length in PcCo^II^–O_2_ is 1.87 Å, with a bonding energy of −0.87 eV, which could be viewed as a moderately strong interaction between the O_2_ and Co catalytic site at room temperature. In contrast, the Ni–O and Cu–O bond lengths are 2.62 and 2.51 Å, respectively, with a very weak bonding energy of −0.01/−0.23 eV (Fig. S58). Such bonding could be easily destroyed by random collisions from solvent molecules that are induced by the thermal vibration even at room temperature. As a result, the situation of the O_2_ molecule in USTB-28–Co is completely different from that of the other two COFs analogues. In USTB-28–Co, the O_2_ molecule could be stabilized at the Co catalytic site, waiting for the beginning of the catalytic cycle that is induced by the random photon capture. In contrast, for USTB-28–M (M=Ni, Cu), the O_2_ molecule could only occasionally locate at the Ni/Cu catalytic site and be ready to leave again at any time, significantly limiting the subsequent occurrence of NHPI oxidation. Further electronic structural calculations also support that USTB-28–Co is the best candidate for binding O_2_ among these three COFs. As shown in Fig. 5c, the electron density is concentrated between the O and Co atoms of PcCo^II^–O_2_, showing the strong O–Co coordination. However, this is not true for USTB-28–M (M=Ni, Cu). In addition, the projected crystal orbital Hamilton population (pCOHP) and partial density of states (pDOS) calculations clearly describe the obvious Co(3d)–O(2p) coordinating mechanism with an absolute integrated crystal orbital Hamilton population value of 1.35 eV, which is obviously larger than the O–Ni/O–Cu coordinating linkages of 0.11/0.07 eV in USTB-28–M (M=Ni, Cu) (Figs S59‒S62). This illustrates that the stable linking between the O_2_ and Co site is the dominating factor to enhance the photocatalytic efficiency of USTB-28–Co. Furthermore, the experimental results prove that the USTB-28–Co's catalytic efficiency is not only higher than that of USTB-28–M (M=Ni, Cu), but also higher than that of the monomeric CoPcF_16_. Theoretical calculations prove the auxiliary function of HHTC in the photon-capturing and *e*^−^*-generating processes. In addition, the well-dispersed metallophthalocyanine active centers in the 3D framework without any aggregation, in combination with the unique porous structures (big pore size and high surface area) of **tbo** topological COFs and trapped PINO, enable this system to have better activity than monomeric phthalocyanine under the same synergistic photocatalytic conditions.

When we look back to the whole reaction mechanism, it is obvious that USTB-28–Co has four advantages in the field of photon-induced EB→AOPH reactions: (i) the medium Co–O bonding strength enables the substrate-O_2_ complex to resist the thermal vibration at room temperature, although it is not too strong to prevent the C–O split in substrate–AOPH; (ii) two photon-injecting processes in one catalytic cycle form more aryl alkane radicals and thus efficiently drive the oxidation reaction; (iii) the introduction of HHTC provides a conjugated electronic system for the whole framework and improves the photocatalytic activity; (iv) the unique **tbo** topology of the porous structure provides a rich pore corner to bind NHPI for affording PINO radical together with the enough reacting space for smooth reactant/product transportation in the cooperative photocatalysis.

## CONCLUSION

In conclusion, a series of metallophthalocyanine 3D COFs with **tbo** topology and irreversible bonds have been prepared under the guidance of reticular chemistry. These frameworks display porous structures with robust dioxin-linkage and high surface areas. In particular, a synergistic visible-light photocatalytic system has been built by using a USTB-28–M radical initiator to convert trapped *N*-hydroxyphthalimide into active phthalimide-*N*-oxyl radical, enabling the aerobic oxidation of various alkyl benzenes into valuable ketones with high yield and selectivity. Mechanism investigation clearly reveals that the trapped phthalimide-*N*-oxyl radical precursor, the unique photophysical behaviors, well-dispersed active cobalt phthalocyanine, large pore size and high surface area of USTB-28–Co play dominant roles in the synergistic photocatalytic system. The present results not only show the first robust functional **tbo** topological COFs with permanent porosities, but also establish a synergistic photocatalysis paradigm by using 3D COF radical initiators towards widening their applications.

## Supplementary Material

nwae396_Supplemental_File
