# Supplementary material for: Robust dioxin-linked metallophthalocyanine tbo topology covalent organic frameworks and their photocatalytic properties
Source: Natl Sci Rev. 2024 Nov 6;12(1):nwae396. doi: 10.1093/nsr/nwae396 (PMC11740510; doi:10.1093/nsr/nwae396)
Supplement: nwae396_Supplemental_File [file nwae396_supplemental_file.pdf]

Robust Dioxin-linked Metallophthalocyanine tbo Topology Covalent Organic Frameworks and Their Photocatalytic Properties

Yucheng Jin,[1] Qianjun Zhi,[1] Hailong Wang,[1,*] Xiaoning Zhan,[1] Dongdong Qi,[1 **] Baoqiu Yu,[1] Xu Ding,[1] Tianying Wang,[2] Heyuan Liu,[2] Mingxue Tang,[3] Jie Liu,[3] Jianzhuang Jiang[1 ***]

[1]Beijing Advanced Innovation Center for Materials Genome Engineering, Beijing Key Laboratory for Science and Application of Functional Molecular and Crystalline Materials, Department of Chemistry and Chemical Engineering, School of Chemistry and Biological Engineering, University of Science and Technology Beijing, Beijing 100083, China
[2]School of Materials Science and Engineering, China University of Petroleum (East China), Qingdao, Shandong 266580, China
[3]Center for High Pressure Science and Technology Advanced Research, Beijing 100094, China

[*] Correspondence: hlwang@ustb.edu.cn
[**]Correspondence: qdd@ustb.edu.cn
[***]Correspondence: jianzhuang@ustb.edu.cn

Contents

Experimental section S3
Figure S1. PXRD of the materials obtained under synthetic conditions S8
Figure S2-4. PXRD patterns of USTB-28-M in different solutions for 1 week S9
Figure S5-7. TGA date of USTB-28-M in N_2 atmosphere. S10
Figure S8. The structure models of USTB-28-M S12
Figure S9. Experimental PXRD pattern of USTB-28-Co S12
Figure S10. Pore size distribution of USTB-28-M S13
Figure S11-13. FT-IR spectra of the USTB-28-M S14
Figure S14. Solid-state ^{19}F NMR spectrum of the USTB-28-Ni S15
Figure S15. SEM images of the USTB-28-M S16
Figure S16. TEM, HRTEM, and EDX mapping images of USTB-28-M S16
Figure S17. X-ray photoelectron spectroscopy survey spectra of USTB-28-M S17
Figure S18. Co 2p XPS spectrum of USTB-28-Co S17
Figure S19. Ni 2p XPS spectrum of USTB-28-Ni S18
Figure S20. Cu 2p XPS spectrum of USTB-28-Cu S18
Figure S21-23. XANES of USTB-28-M S19
Figure S24. PXRD data of CoPc-O-COF S20
Figure S25. PXRD patterns of USTB-28-M S21
Figure S26-28. FT-IR spectra of USTB-28-M S21
Figure S29. TEM photos of USTB-28-M S23
Figure S30. HRTEM photos of USTB-28-M S23
Figure S31-36. FT-IR spectra of the NHPI and USTB-28-M S24
Figure S37-38. Different interaction in USTB-28-M and NHPI. S27
Figure S39. FT-IR spectra of the NHPI and $CoPcF_{16}$. S28
Figure S40-41. Different interaction in CoPc-O-COF and NHPI. S28
Figure S42. UV−vis-DRS S29
Figure S43-45. The value of band gaps of USTB-28-M S30
Figure S46-48. MS curves of USTB-28- M S31
Figure S49. Band gaps of USTB-28-M S33
Figure S50. Photocurrents under Xe lamp irradiation of USTB-28-M S33
Figure S51-52. EPR data of USTB-28-M S34
Figure S53. The kinetic trace of USTB-28-M, $CoPcF_{16}$, and CoPc-O-COF S35
Figure S54-56. The fs-TA spectra of the USTB-28-Co in different conditions S35
Figure S57. π-LOL of USTB-28-M S37
Figure S58-62. Calculated oxygen adsorption on the Metal atom S37
Table S1. Synthetic conditions screened for the synthesis of USTB-28-Co S40
Table S2. The fitting parameters for EXAFS data for USTB-28-M S41
Table S3. The tested ICP-OES results for USTB-28-M S41
Table S4. Elemental analysis of USTB-28-M S41
Table S5-7. Photocatalytic oxidation of benzylic under different conditions S42
Table S8. Comparison of catalytic performances with various catalysts. S44
Reference S45

1. Experimental section

1.1 Reagents

All the starting materials were purchased from TCI or Aladdin and used without further purification. *N*-Methylpyrrolidone (NMP), mesitylene (Mes), *N,N*-Dimethylformamide anhydrous (DMF), trimethylamine (Et_3N), dichloromethane (DCM), petroleum ether (PE), dichlorobenzene, ethyl acetate, toluene, *n*-butanol, tetrahydrofuran (THF), acetic acid, and methanol were purchased from Admas Reagent, compounds $MPcF_{16}$ (M = Co, Ni, Cu) were purchased from Jilin Chinese Academy of Sciences Yanshen Technology Co., Ltd. HHTC was prepared according to the reported literature.[1]

1.2 Synthesis

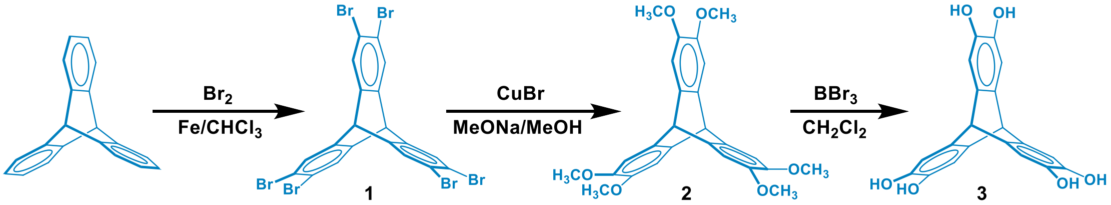

Scheme S1. Chemical synthesis of the 2,3,6,7,14,15-hexahydroxyltriptycene (3, also named HHTC).

Synthesis of 2,3,6,7,12,13-hexabromotriptycene (1)

To a solution of triptycene (3.00 g, 11.80 mmol) in chloroform (200 mL) was added iron powder (85.0 mg, 1.52 mmol) at room temperature under vigorous stirring. The mixture was stirred for 15 min, then bromine (3.8 mL, 73.84 mmol) in chloroform (20 mL) was added in one portion. The reaction mixture was then vigorously stirred under reflux for 130 min, cooled to room temperature and the solvent was removed under reduced pressure. The resulting brown powder was redissolved in chloroform (*ca.* 500 mL) and filtered through a pad of silica gel using additional chloroform (*ca.* 200 mL). The chloroform was then removed under reduced pressure and the resulting yellow solid was dissolved in hot acetone (800 mL, 52 ^oC). The acetone solution was then cooled to 0 ^oC in an ice bath to give 6.33 g crude **1**. The crude product (6.33 g) was again dissolved in acetone (700 mL, 52 ^oC) and cooled to 0 ^oC to give pure **1** (2.51 g, 29 %) as colorless crystals. The mother liquor was concentrated to *ca.* 200 mL and cooled again to give additional **1** (2.48 g, 29 %). ^1H NMR (400 MHz, $CDCl_3$) δ (ppm): 5.23 (s, 2H), 7.62 (s, 6H).

Synthesis of 2,3,6,7,12,13-hexamethoxytriptycene (2)

The mixture of 2,3,6,7,12,13-hexabromotriptycene (**1**) (4.92 g, 6.76 mmol), copper(I) bromide (0.60 g, 4.20 mmol), 25 wt% sodium methoxide in methanol (70.0 mL, 1.22 mol), ethyl acetate (5.0 mL), and toluene (100.0 mL) was refluxed under nitrogen for 20 h. The solution was cooled and quenched by the addition of water

(50.0 mL). After extraction of the aqueous layer with dichloromethane, the combined organic layers were dried over anhydrous $MgSO_4$ and filtered. The solvent was removed by rotary evaporation to give the desired product as a white powder (2.72 g, 93%). ^{1}H NMR (400 MHz; $CDCl_3$) δ (ppm): 3.84 (s, 18H), 5.19 (s, 2H), 7.01 (s, 6H).

Synthesis of 2,3,6,7,14,15-hexahydroxyltriptycene (3)

Boron tribromide (3.40 g, 19.80 mmol) was added dropwise to a stirred solution of 2,3,6,7,12,13-hexamethoxytriptycene (**2**) (1.48 g, 3.0 mmol) in dry dichloromethane (70.0 mL) at 0 ^{o}C under a nitrogen atmosphere and then the reaction mixture was stirred at room temperature for 3 h. After pouring onto a mixture of ice and water, the precipitate was collected by filtration, washed with cold water and dried. The crude product was recrystallized from THF/petroleum ether (40-60 ^{o}C) to give pale brown crystals (1.20 g, 98 %). ^{1}H NMR (400 MHz; CD_3OD) δ (ppm): 1.60 (t, 6H), 2.71 (q, 4H), 6.80 (s, 6H), 7.10 (s, 6H).

Synthesis of USTB-28-Co, USTB-28-Ni and USTB-28-Cu

$CoPcF_{16}$ (8.6 mg, 0.010 mmol) and HHTC (4.7 mg, 0.013 mmol) were added into 0.35 mL NMP and 0.65 mL Mes in a 10.0 mL Pyrex tube. The mixture was sonicated for 15 min to form a homogeneous suspension. Then 200.0 μL Et_3N was added into the mixture. After three freeze-pump-thaw cycles, the Pyrex tube was sealed and heated in an oven at 180 ^{o}C for 7 days. The black-green precipitate was collected by centrifugation and rinsed with THF in a Soxhlet extractor for one week. The resulting COF was obtained as black powder in a yield of 74%. By means of the synthesis procedure for HHTC with $NiPcF_{16}$ (8.6 mg, 0.010 mmol) or $CuPcF_{16}$ (8.6 mg, 0.010 mmol) instead of $CoPcF_{16}$, USTB-28-Ni and USTB-28-Cu were obtained as black-green powder in a yield of 71% and 79%, respectively.

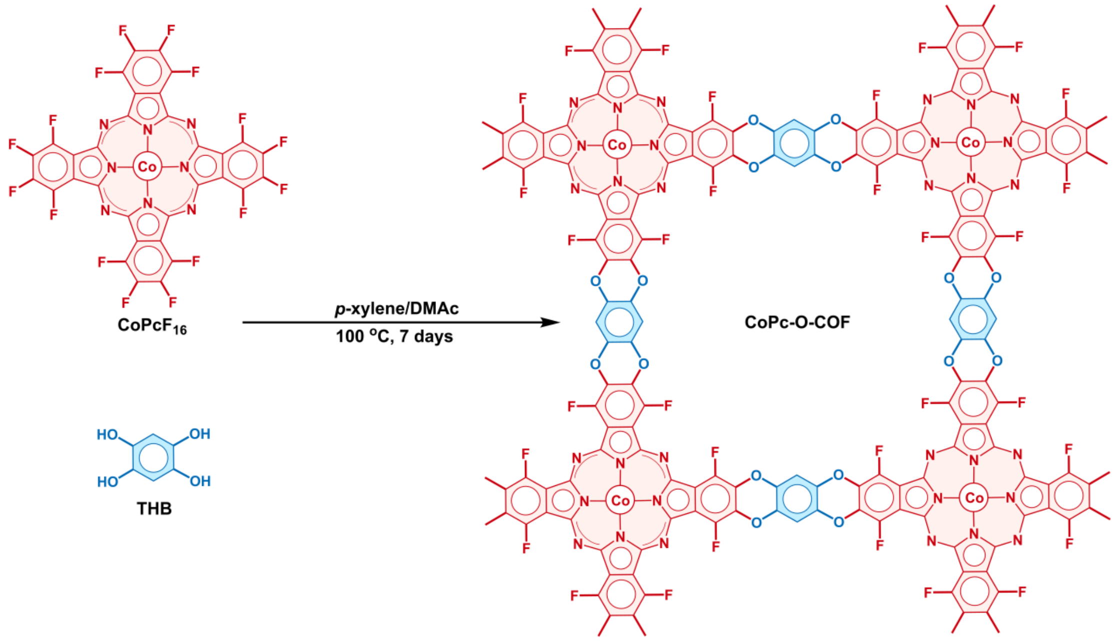

Scheme S2. Synthesis and structure of CoPc-O-COF.

Synthesis of CoPc-O-COF

$CoPcF_{16}$ (8.59 mg, 0.010 mmol) and THB (2.84 mg, 0.020 mmol) were added into the mixed solvent of 0.7 mL p-xylene and 0.5 mL DMAc in a 10 mL Pyrex tube. The mixture was sonicated for 15 min to form a homogeneous suspension. Then 100 μL triethylamine was added into the mixture. After three freeze-pump-thaw cycles, the Pyrex tube was sealed and heated in an oven at 100 ℃ for 7 days. The black-green precipitate was collected by centrifugation and rinsed with acetone, dichloromethane, and THF in a Soxhlet extractor for one day. Finally, CoPc-O-COF was then obtained as black powder in a yield of 75%.

1.3 Characterizations

Powder X-ray diffraction (PXRD) analyses were performed using a PANalytical Empyrean diffractometer operating at 45 kV voltage and 40 mA current with Cu-Kα X-ray radiation (λ = 0.154056 nm). Fourier transform infrared (FT-IR) spectra were measured by a Bruker Tensor 37 infrared spectrometer. The solid-state ^{19}F NMR spectrum was recorded on a 400MHz WB Solid-State NMR Spectrometer. Scanning electron microscopy (SEM) images were obtained by a SU8010 electron microscopy. Transmission electron microscopy (TEM) images were collected from a HT7700 electron microscope at 100 KV. High-resolution TEM (HRTEM) were collected by transmission electron microscopy (JEM-2200FS) at an operation voltage of 200 kV. Thermogravimetric analysis (TGA) was characterized by SDTA851e thermo-analyzer. N_2 adsorption-desorption isotherms were measured on a ASAP 2020 PLUS HD88 apparatus at 77 K and the surface areas were calculated by the Brunauere Emmette Teller (BET) method. Ultraviolet-visible diffuse reflectance absorption spectrum (UV-vis-DRS) absorption spectra were collected using a Shimadzu UV-2600 UV-vis spectrophotometer. The analysis ofvarious aryl halides was conducted on a gas chromatography-mass spectrometry (GC-MS, Trace 1300-ISQ, Thermo Fisher) coupled with a CP-WAX 52 CB (0.25 μm × 0.5 mm × 30 m) capillary column. The analysis was performed as follows: sample injection volume 0.5 μL, inlet temperature 250 ^oC, carrier gas flow rate 1.0 mL/min, splitting ratio 100, ion source temperature 230 ℃, mass spectrometry scan range 45-450 m/z. The initial temperature of the oven was 80 ℃, kept for 1 min, then heated up to 250 ^oC at a rate of 20 ^oC /min and kept for 5 min. Electron paramagnetic response (EPR) spectra were obtained using a Bruker ELEXSYS II E 500 EPR Spectrometer and Bruker BIOSPIN GMBH EMX nano at room temperature.

1.4 Photocatalytic oxidation of arylalkanes

Arylalkane (0.3 mmol), USTB-28-M (0.002 mmol based on MPc unit) and *N*-hydroxyphthalimide (NHPI) (0.02 mmol) in acetonitrile (2.0 mL) were stirred at room temperature under the irradiation of a 350 W Xe lamp for 8 h in the presence of O_2. The solid was recovered by centrifugation, and thoroughly washed with MeOH, THF and CH_3CN several times. The recovered solid was reused in the successive run. The

identity of the product was determined by GC analysis. The turnover frequency (TOF, h^{-1}) is calculated by using the equation:
TOF = Amount of acetophenone evolved/(amount of active sites in catalyst*time)

Study of the apparent quantum yield (AQY) for the photocatalytic oxidation of alkyl benzenes by USTB-28-Co: A 300 W Xe lamp with monochromatic light filter and optical power meter (PL-MW2000) was used to collect the apparent quantum yield (AQY), which was defined as the ratio between the number of collected carriers and the number of all incident photons on the device active area at a given wavelength. AQY was calculated according to the following equation:

$$\text{AQY (\%)} = \frac{n \cdot N_A \cdot h \cdot c}{I \cdot \lambda \cdot A \cdot t} * 100$$

Where n is the moles of photogenerated product, N_A is the Avogadro's constant (6.02 $\times 10^{23}$ mol^{-1}), h is the Planck constant (6.63 $\times 10^{-34}$ J s), c is the speed of light (3 $\times 10^{8}$ m s^{-1}), I is the light intensity, λ is the wavelength of incident monochromatic light, A is the cross-sectional area of irradiation, and t is the irradiation time.

1.5 Photocurrents and Mott-schottky (MS) curves measurements

2.0 mg of catalysts were dispersed in a mixture containing 0.5 mL ethanol and 10.0 μL Nafion solution (5 wt%) and ultrasonic treated to form homogenous catalyst ink. Then the catalyst ink was dipped on a polished FTO glass and dried in air. These measurements were conducted on a CHI 760E electrochemical work station in a three-electrode cell system under irradiation of a 300 W Xe lamp (Perfect Light PLS-SXE 300+) with a 400 nm cutoff filter. The cycle FTO glass (3.14 × 0.25 × 0.25 cm^2) deposited with materials was used as the photoelectrode, a Pt foil was used as the counter electrode, and Ag/AgCl electrode was used as the reference electrode. The three electrodes were inserted in a quartz cell filled with 0.2 M Na_2SO_4 electrolyte. The Na_2SO_4 electrolyte was purged with N_2 for 1 h prior to the measurements.

1.6 Experimental methods of transient absorption spectra (Fs-TA)

TA spectra were recorded on a femtosecond pump–probe detection system. The laser is generated from an ultrafast laser amplifier (Coherent, central wavelength: 500 nm, repetition rate: 1 kHz, pulse width: 100 fs, pulse energy: 6 mJ). The output laser is divided into two beams of light with a beam splitter. The reflected light is applied to a pump optical parametric amplifier (OPA, Spectra-physics, TOPAS) to generate a pump beam of different wavelengths (250–2500 nm). The pump beam is converted to 500 Hz by a chopper, which is used to excite the sample, and its intensity is tuned via a continuously variable neutral density filter wheel. The transmitted light was used to generate the continuous white light (350–800 nm) via a CaF_2 crystal and acted as the probe beam. Then, the probe beam is collected by a fiber-coupled spectrometer after the sample. Using an optical delay line, the delay between the pump and probe beams is controlled. The samples of $CoPcF_{16}$ and USTB-28-Co were dispersed in CH_3CN. For all fs-TA measurements, samples were filled in 1 mm airtight cuvettes prepared in

a N_2-filled glove box and measured under ambient conditions, and the UV-visible (UV-vis) absorbance of all samples was adjusted to 0.5 before further characterization.

1.7 Electron paramagnetic resonance (EPR).

EPR measurements were performed at X-band (~9.5 GHz) using a Bruker Elexsys E680-X/W EPR spectrometer. The temperature was controlled by a continuous flow cryostat using liquid N_2.

1.8 Theoretical calculation details

In an attempt to evaluate the oxygen adsorption property of USTB-28-M and $MPcF_{16}$, first-principles calculations were carried out via Vienna ab initio package (VASP)[2,3] program by using projected augmented wave (PAW) potential[4] with a cutoff energy of 400 eV. The Perder-Burke-Ernzergof (PBE) functional was used within the formulation of generalized gradient approximation (GGA)[5,6] for the exchange-correlation term. The reciprocal space was sampled by the gamma point in the Brillouin zone with a grid of $2 \times 2 \times 1$. The energy difference of 1.0×10^{-5} eV per atom and the max force of 0.02 eV/Å were set as the convergence criteria for geometry optimization. DFT calculations on fragment model in this study were performed using the Gaussian 16 program.[7] A M06-2X functional with 6-311+G(d,p) basis set.[8-10]

2. Characterizations and Properties

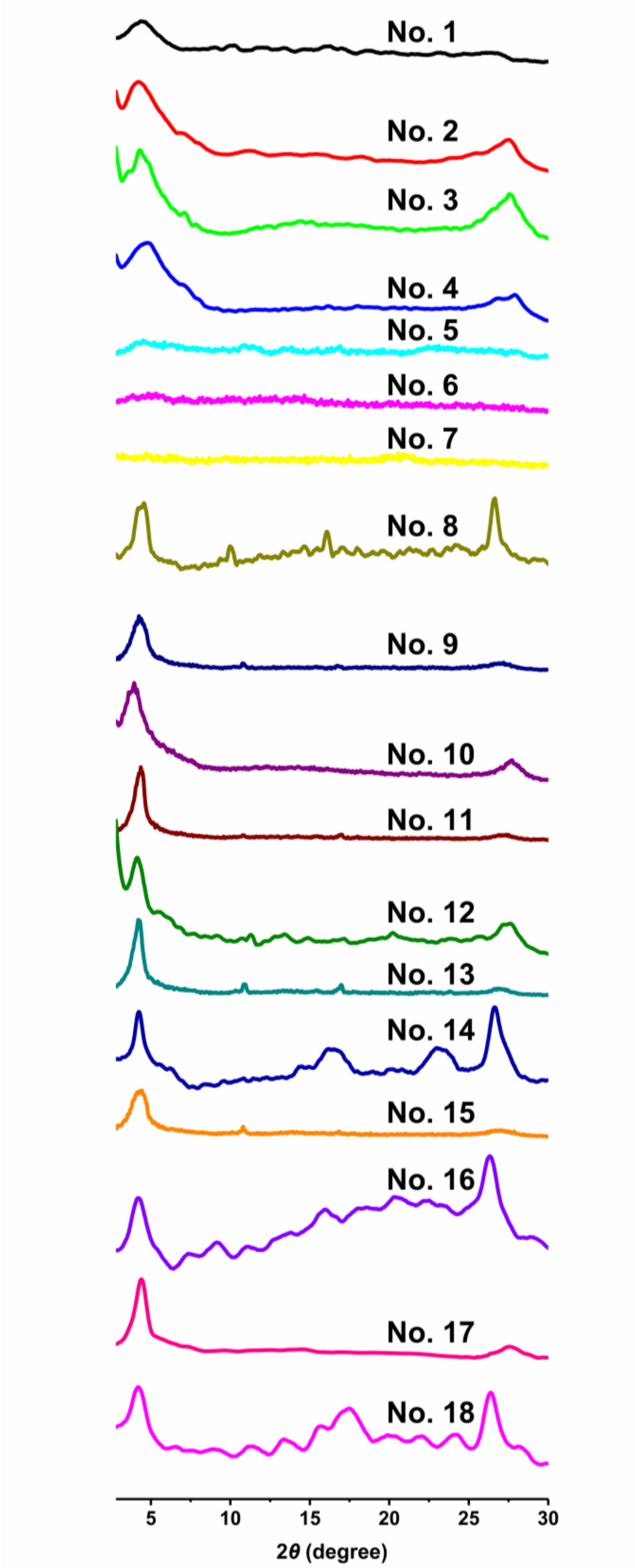

Figure S1. PXRD of the materials obtained under synthetic conditions demonstrated in Table S1.

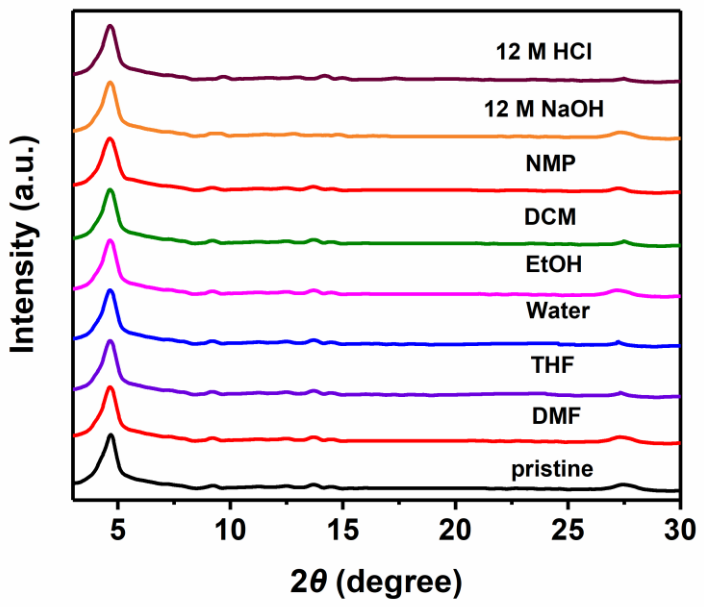

Figure S2. PXRD patterns of USTB-28-Co in different solutions for 1 week.

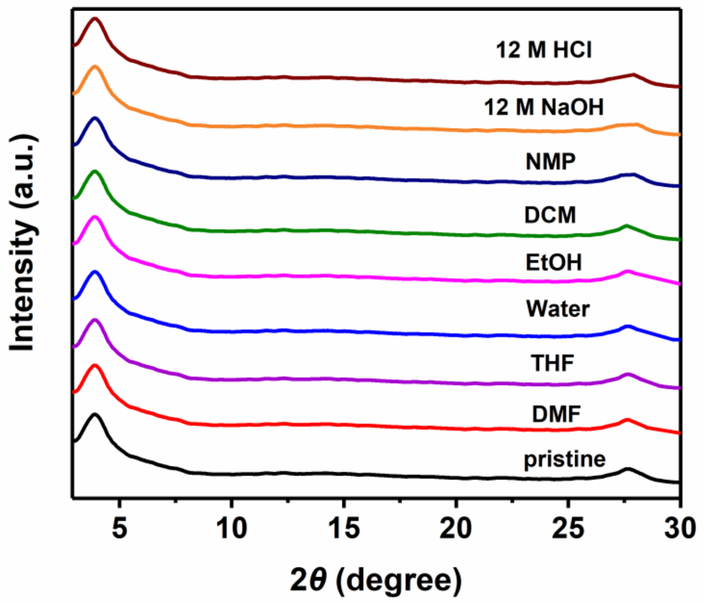

Figure S3. PXRD patterns of USTB-28-Ni in different solutions for 1 week.

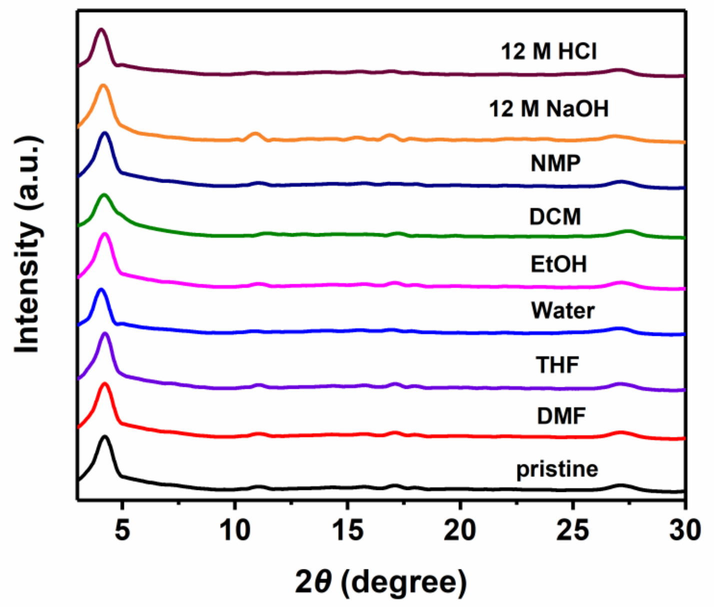

Figure S4. PXRD patterns of USTB-28-Cu in different solutions for 1 week.

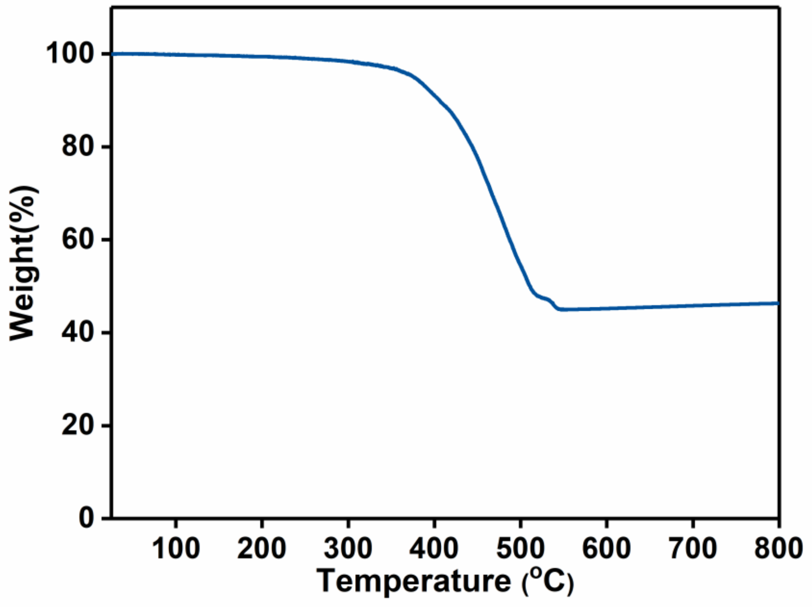

Figure S5. TGA date of USTB-28-Co in N_2 atmosphere.

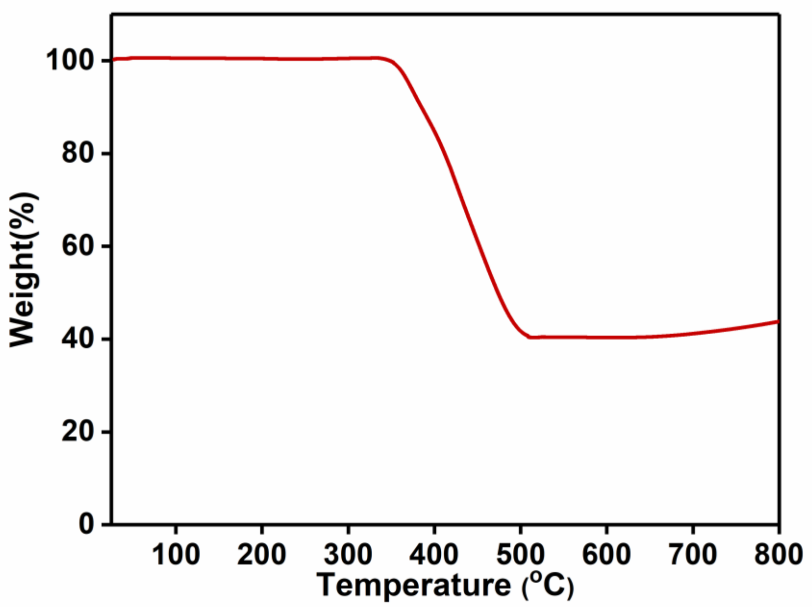

Figure S6. TGA date of USTB-28-Ni in N_2 atmosphere.

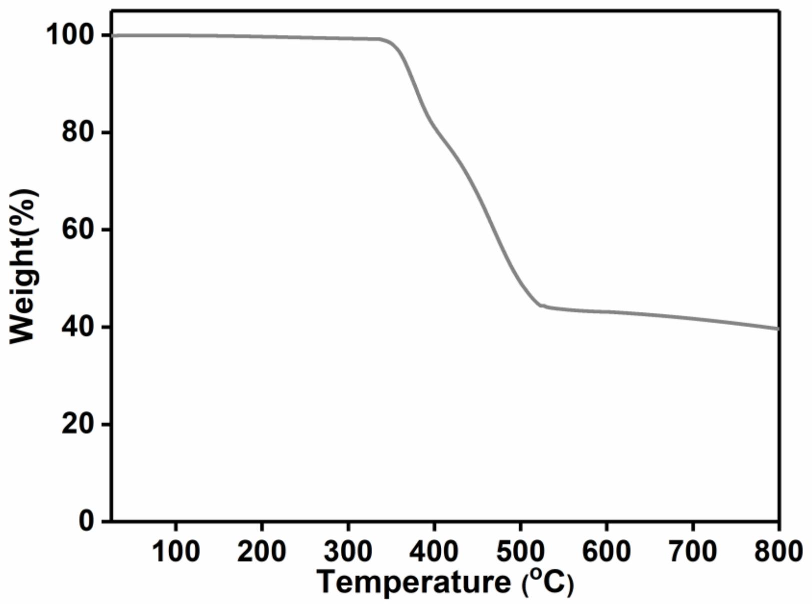

Figure S7. TGA date of USTB-28-Cu in N_2 atmosphere.

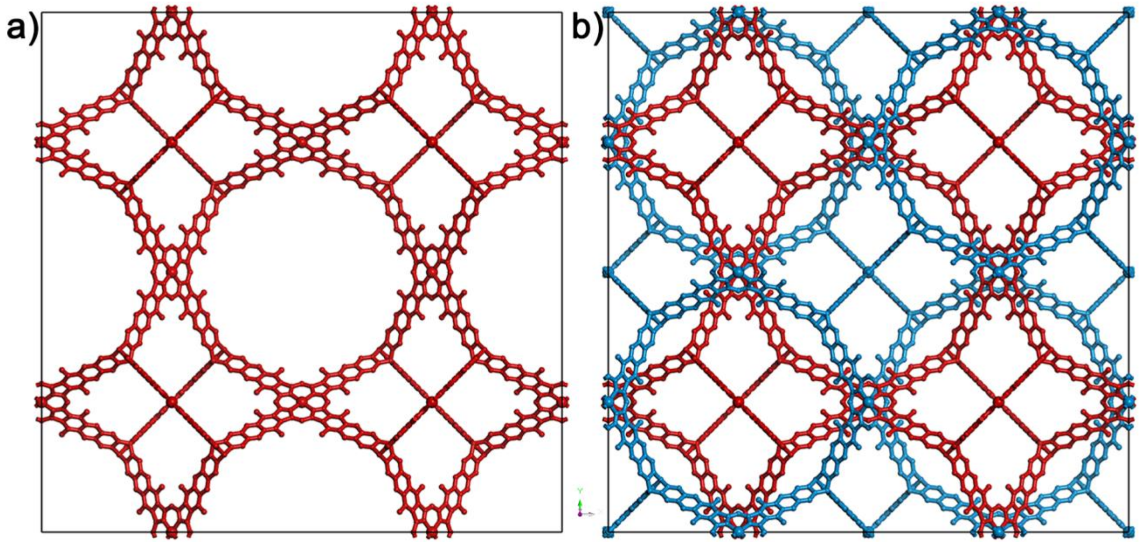

Figure S8. The structure models of USTB-28-M showing the simulated non-interpenetration (a) and 2-fold interpenetration (b) of **tbo** frameworks (different color represents different framework).

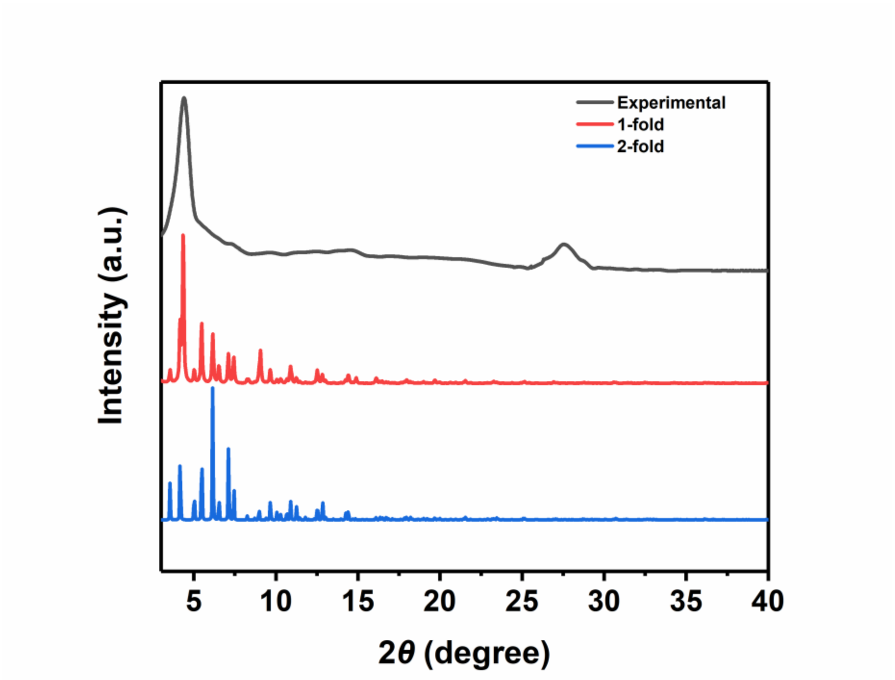

Figure S9. Experimental PXRD pattern of USTB-28-Co (grey), the simulated non-interpenetration (red) and 2-fold (blue) interpenetration **tbo** frameworks.

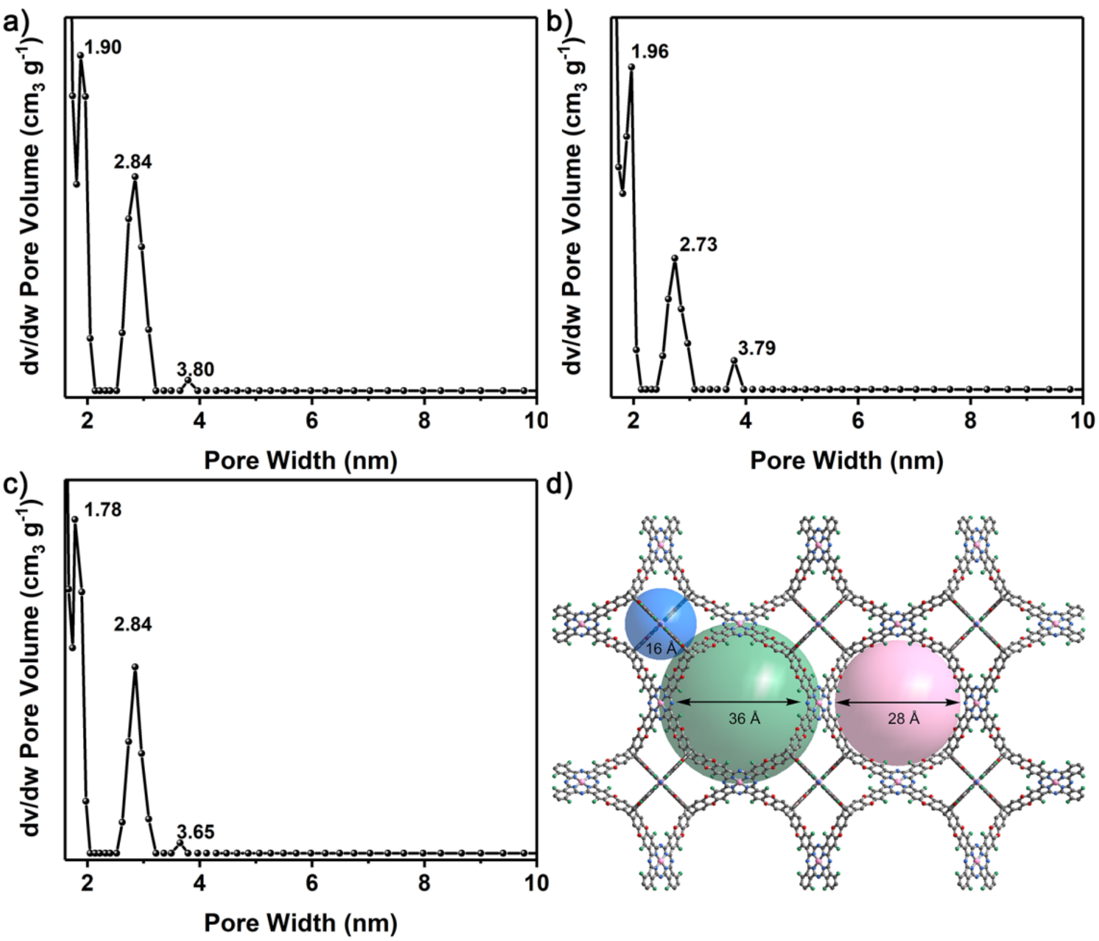

Figure S10. Pore size distribution of (a) USTB-28-Co, (b) USTB-28-Ni, and (c) USTB-28-Cu. (d) Representative section of the crystal structure of USTB-28-Co with the blue, pink, and green balls represent three kinds of voids.

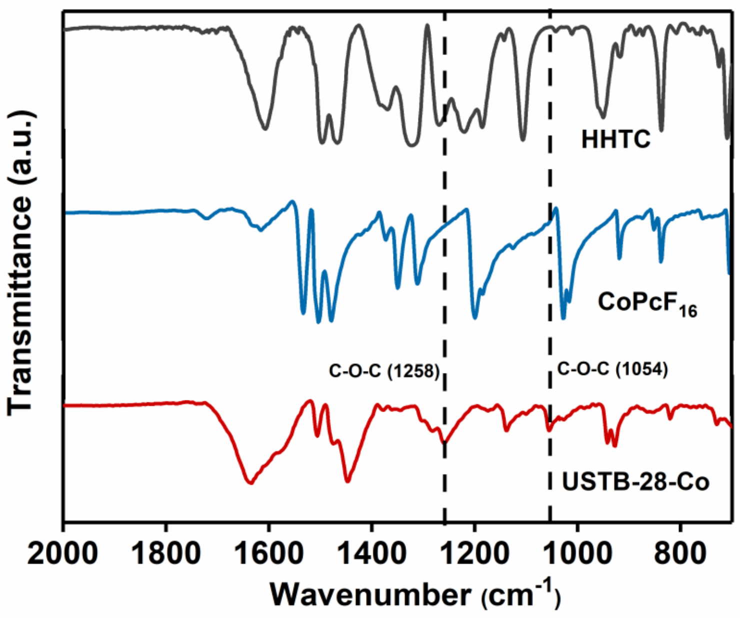

Figure S11. FT-IR spectra of the HHTC, $CoPcF_{16}$, and USTB-28-Co.

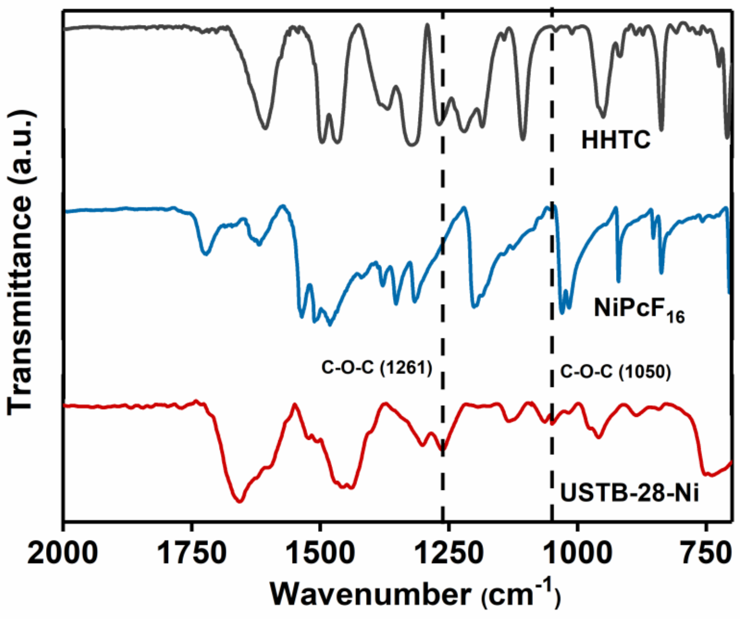

Figure S12. FT-IR spectra of the HHTC, $NiPcF_{16}$, and USTB-28-Ni.

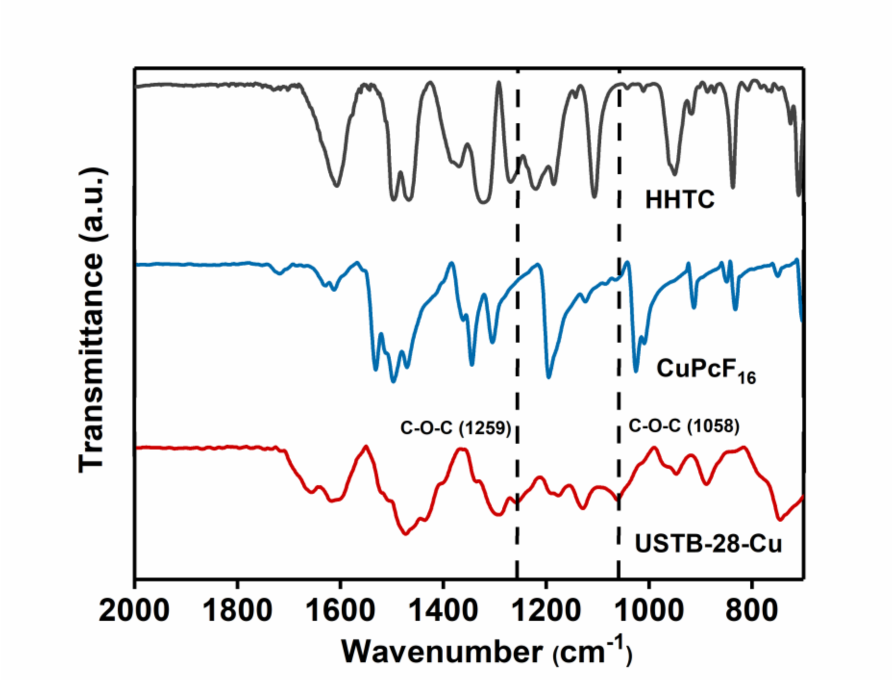

Figure S13. FT-IR spectra of the HHTC, $CuPcF_{16}$, and USTB-28-Cu.

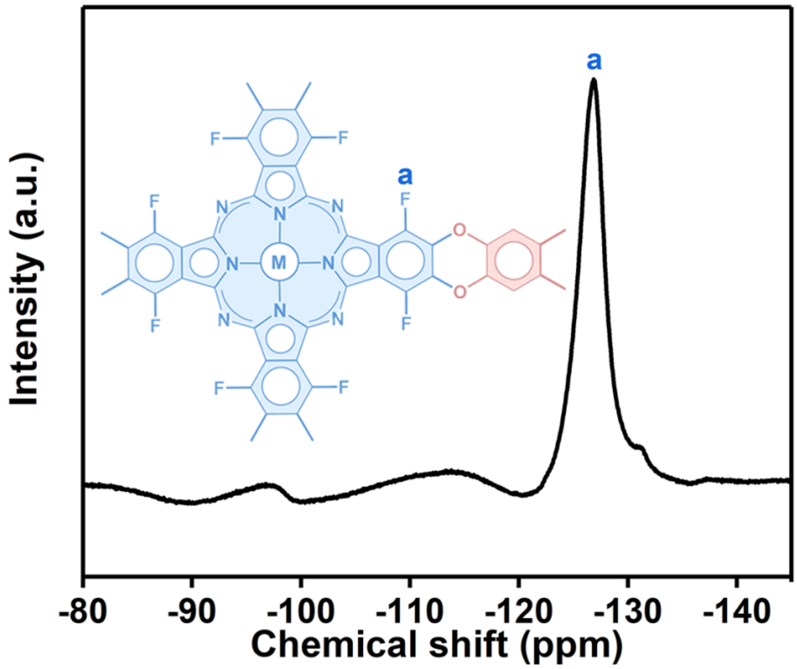

Figure S14. ^{19}F NMR spectrum of the USTB-28-Ni.

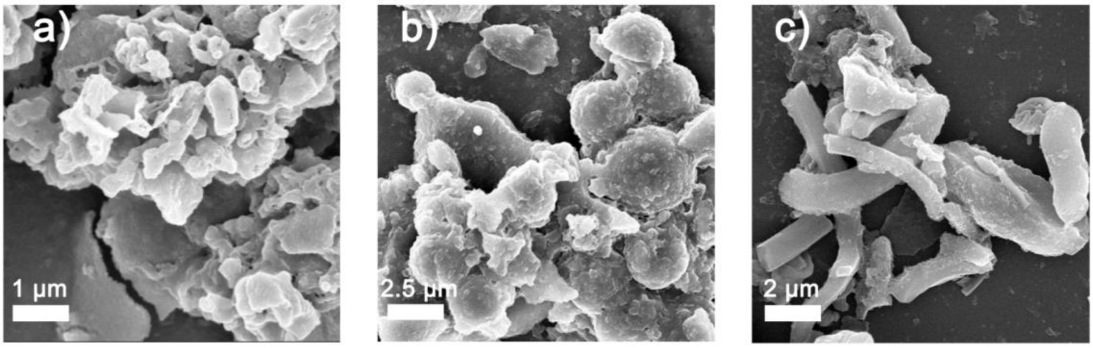

Figure S15. SEM images of the USTB-28-Co (a), USTB-28-Ni (b), and USTB-28-Cu (c).

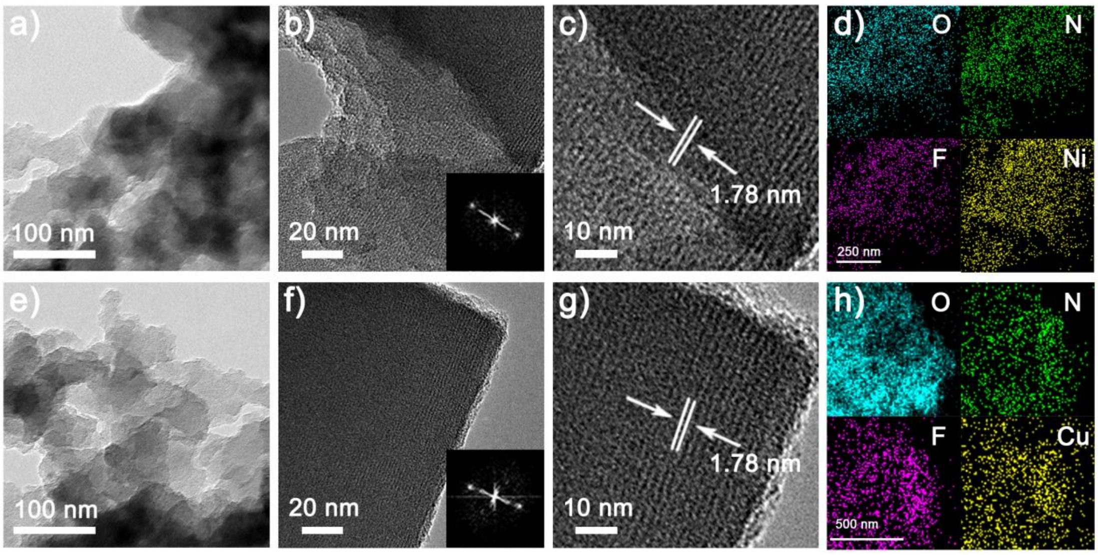

Figure S16. (a) TEM, (b, c) HRTEM, and (d) EDX mapping images of USTB-28-Ni. (e) TEM, (f, g) HRTEM, and (h) EDX mapping images of USTB-28-Cu.

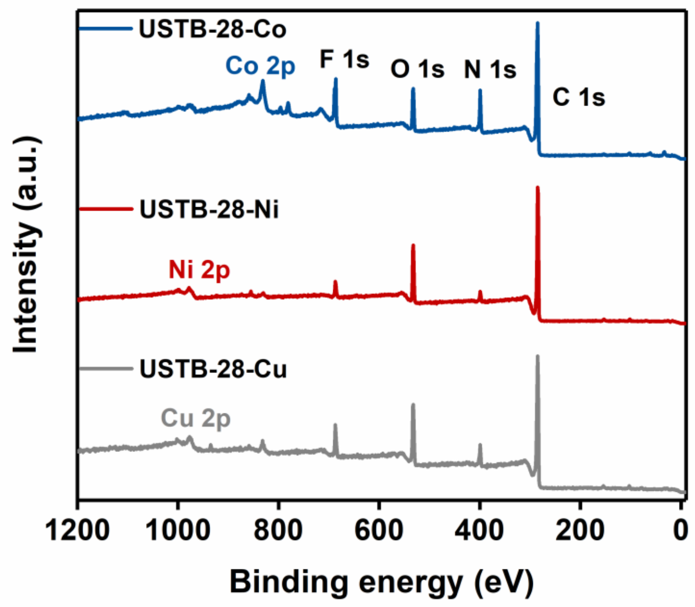

Figure S17. X-ray photoelectron spectroscopy survey spectra of USTB-28-Co, USTB-28-Ni, and USTB-28-Cu.

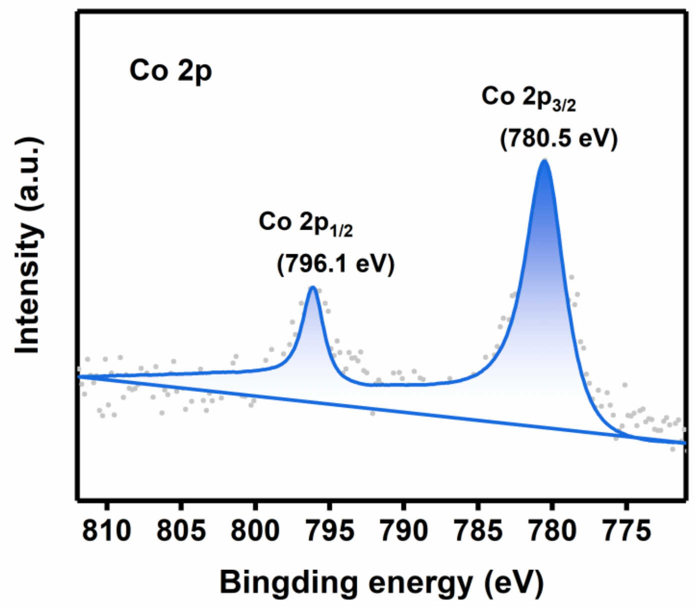

Figure S18. Co 2p XPS spectrum of USTB-28-Co.

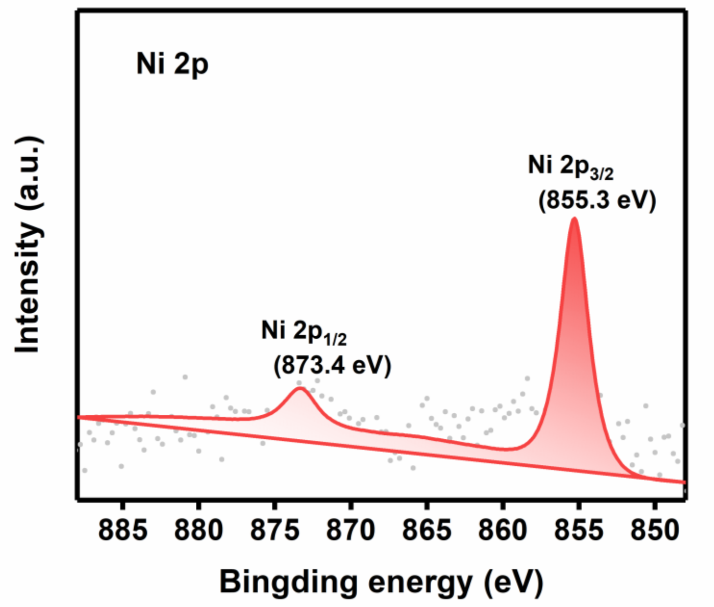

Figure S19. Ni 2p XPS spectrum of USTB-28-Ni.

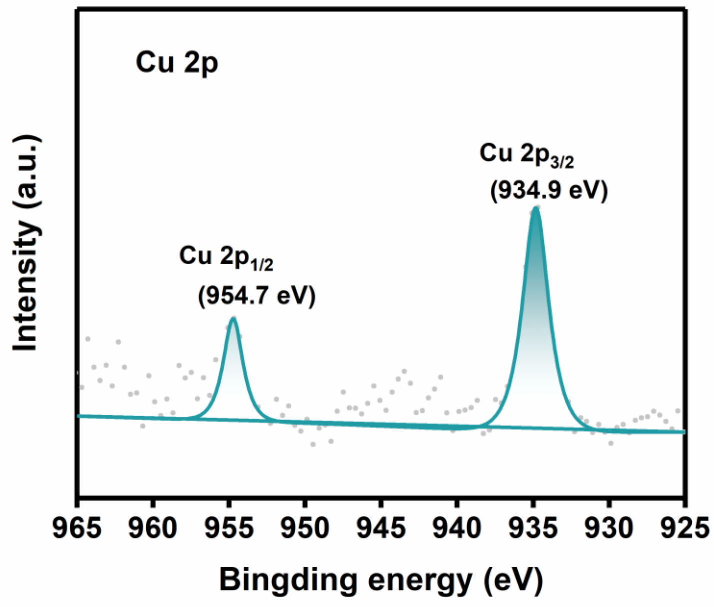

Figure S20. Cu 2p XPS spectrum of USTB-28-Cu.

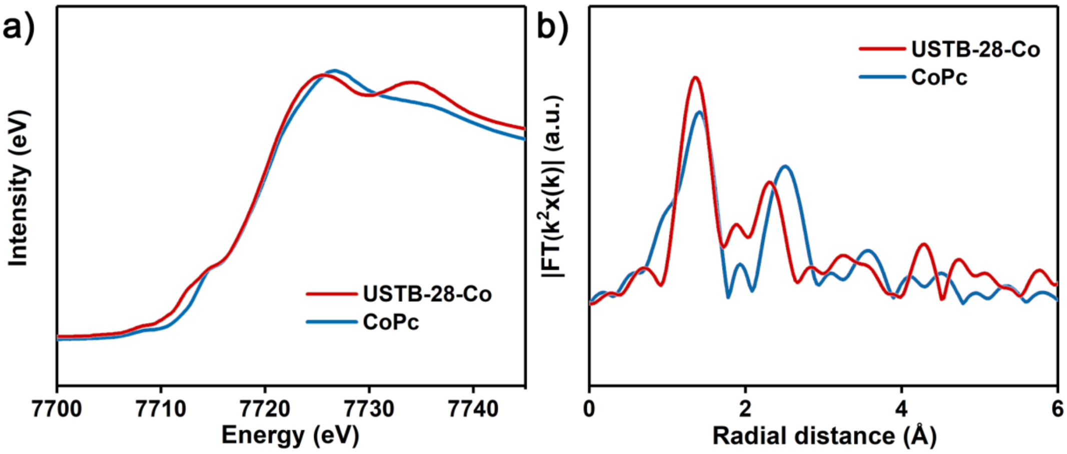

Figure S21. (a) XANES of Co K-edge and (b) EXAFS spectra of CoPc and USTB-28-Co.

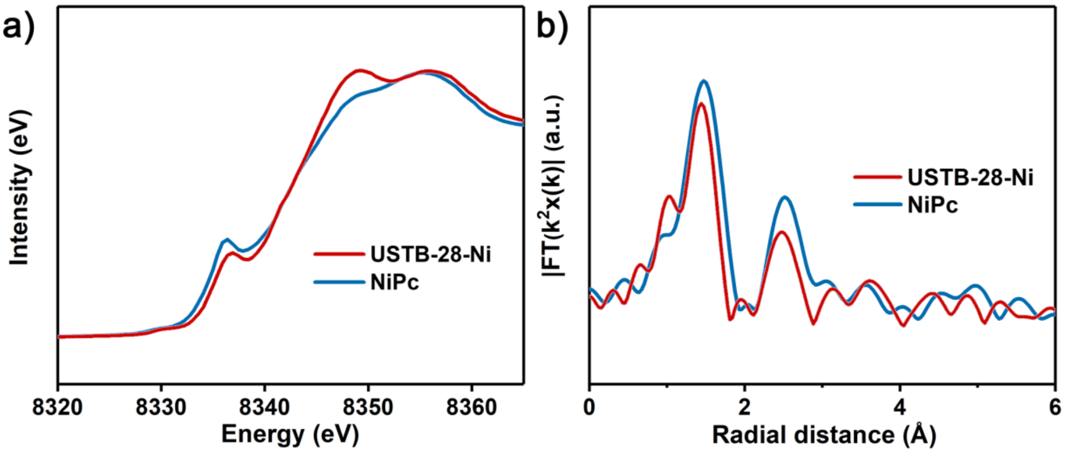

Figure S22. (a) XANES of Ni K-edge and (b) EXAFS spectra of NiPc and USTB-28-Ni.

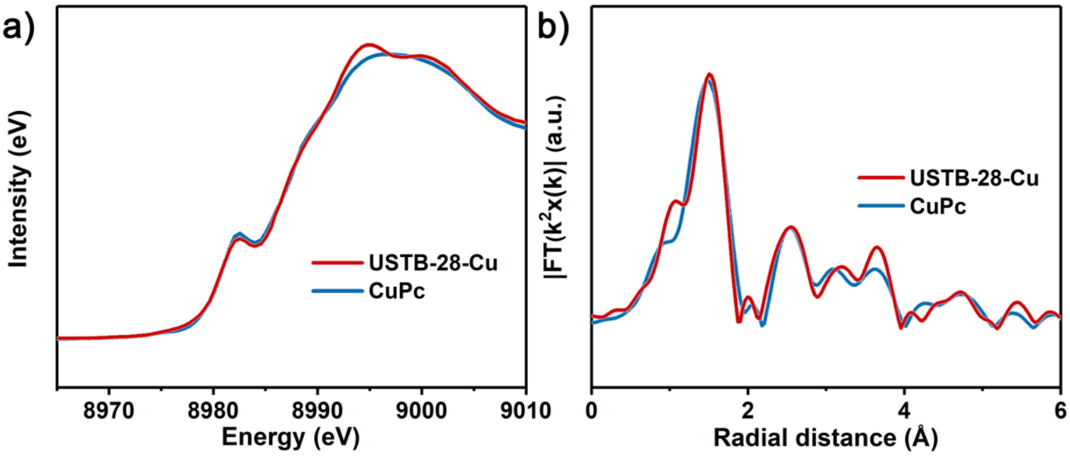

Figure S23. (a) XANES of Cu K-edge and (b) EXAFS spectra of CuPc and USTB-28-Cu.

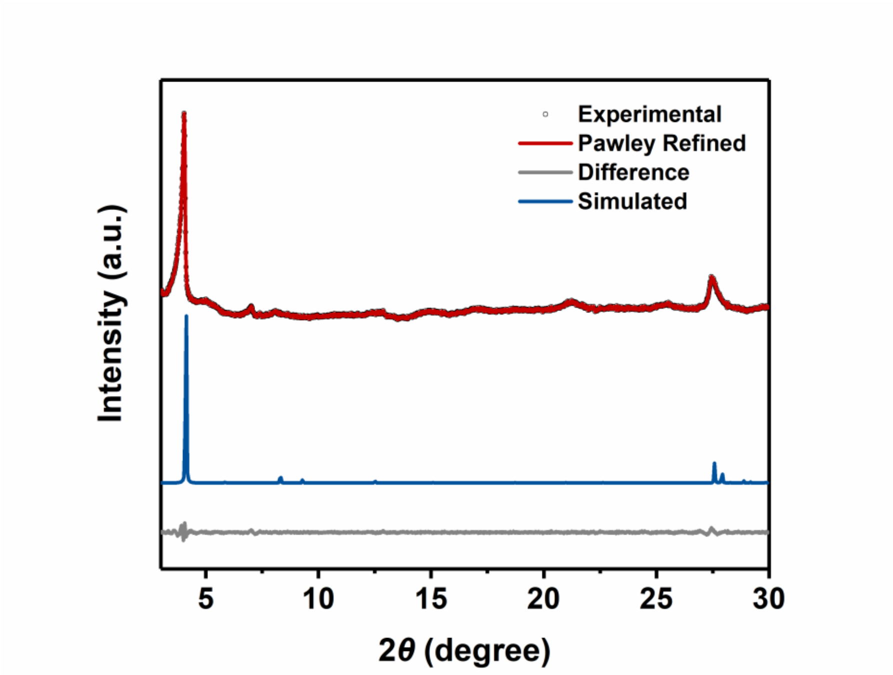

Figure S24. PXRD data of CoPc-O-COF (experimental PXRD profile (black), refined profile (red), simulation pattern (blue), and the difference between the experimental and refined PXRD (grey), respectively.

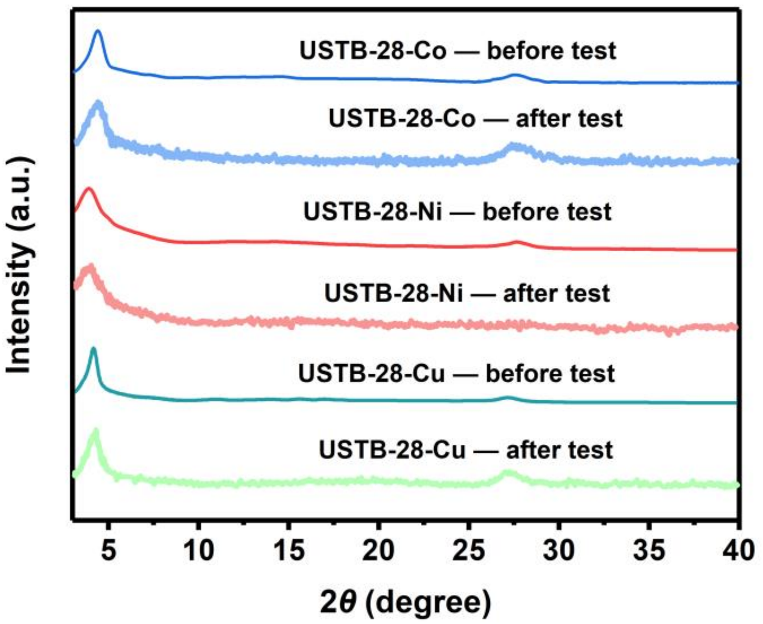

Figure S25. PXRD patterns of USTB-28-M before and after photocatalytic reaction.

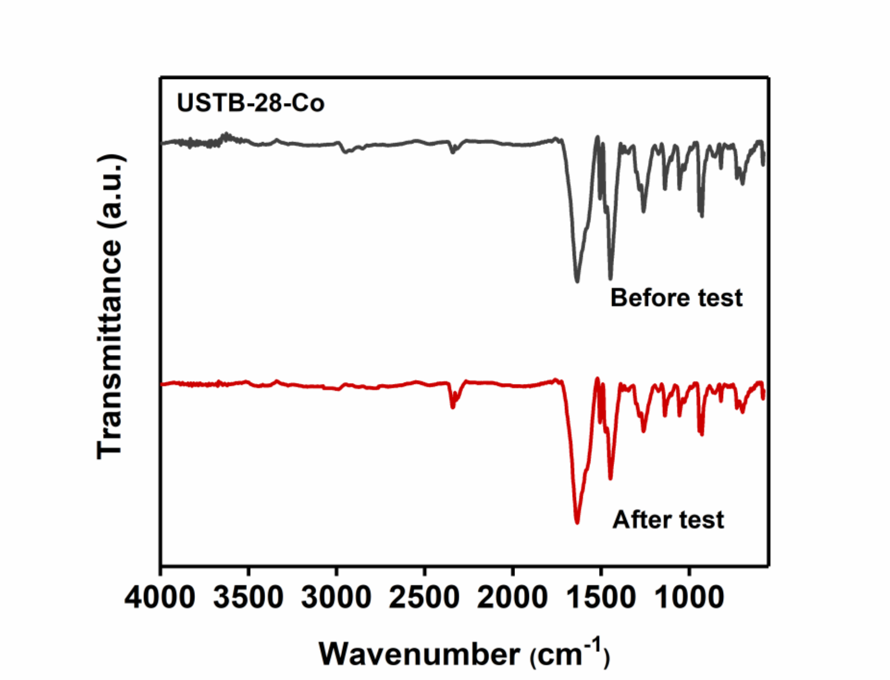

Figure S26. FT-IR spectra of USTB-28-Co before and after photocatalytic reaction.

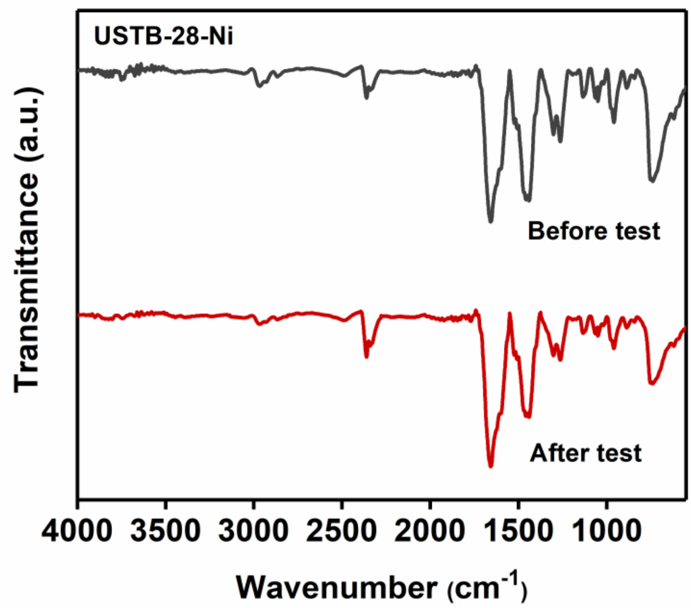

Figure S27. FT-IR spectra of USTB-28-Ni before and after photocatalytic reaction.

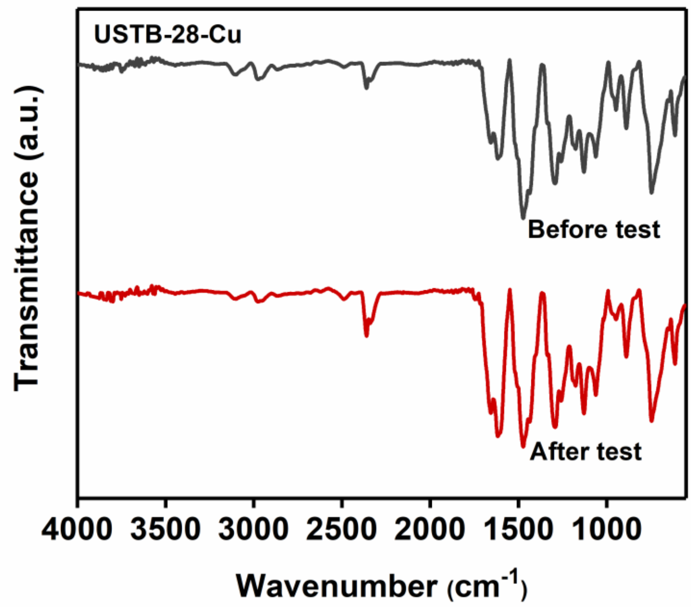

Figure S28. FT-IR spectra of USTB-28-Cu before and after photocatalytic reaction.

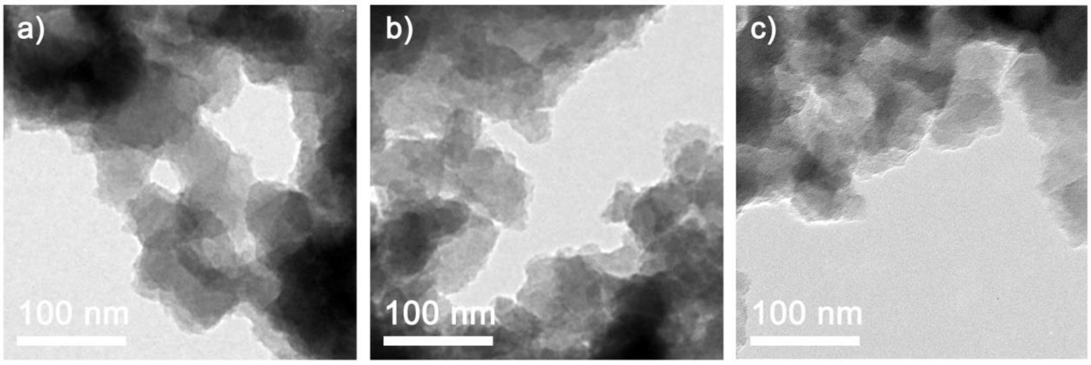

Figure S29. TEM photos of (a) USTB-28-Co, (b) USTB-28-Ni, and (c) USTB-28-Cu after photocatalytic reaction.

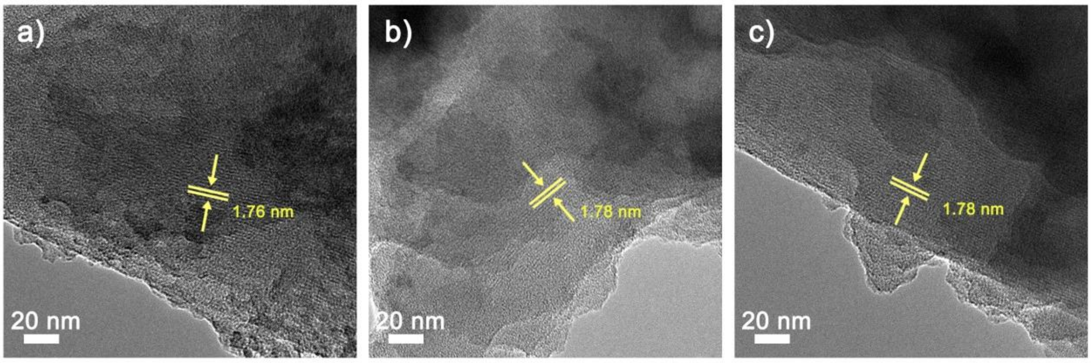

Figure S30. HRTEM photos of (a) USTB-28-Co, (b) USTB-28-Ni, and (c) USTB-28-Cu after photocatalytic reaction.

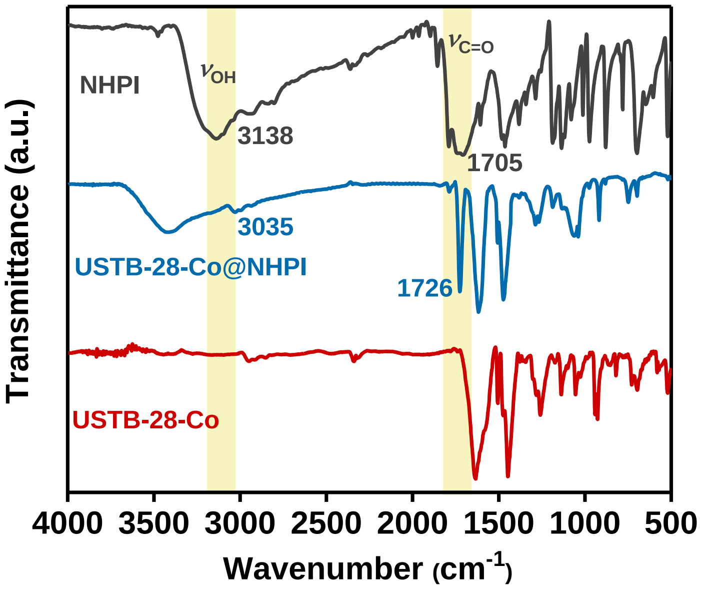

Figure S31. FT-IR spectra of NHPI, USTB-28-Co@NHPI, and USTB-28-Co.

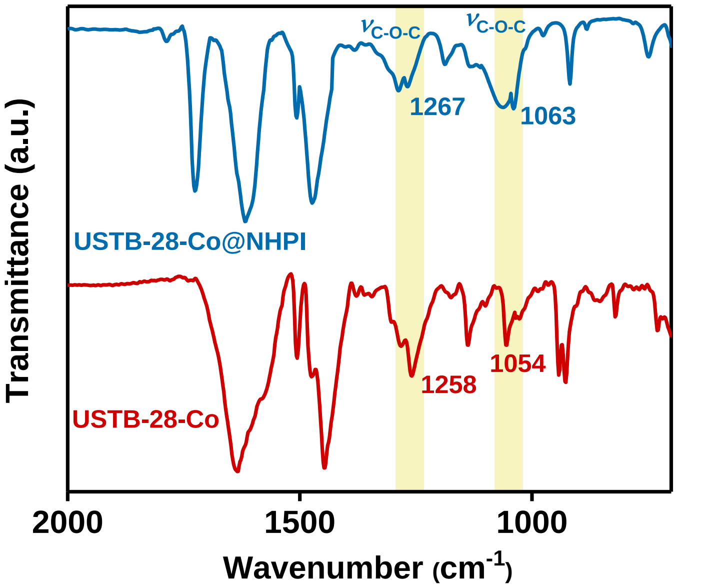

Figure S32. The magnified FT-IR spectra of USTB-28-Co@NHPI and USTB-28-Co.

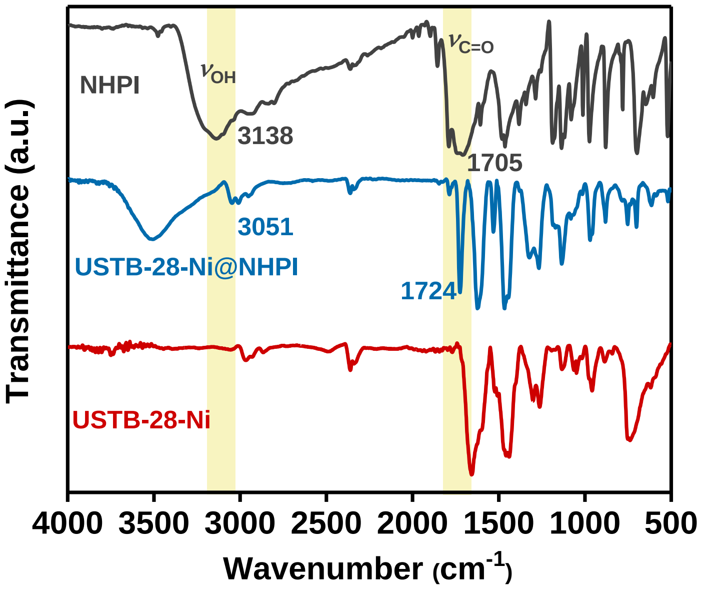

Figure S33. FT-IR spectra of NHPI, USTB-28-Ni@NHPI and USTB-28-Ni.

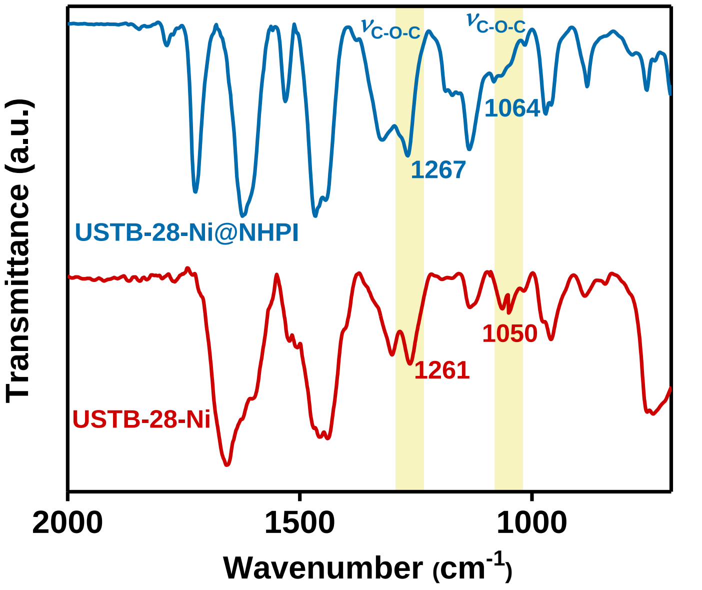

Figure S34. FT-IR spectra of USTB-28-Ni@NHPI and USTB-28-Ni.

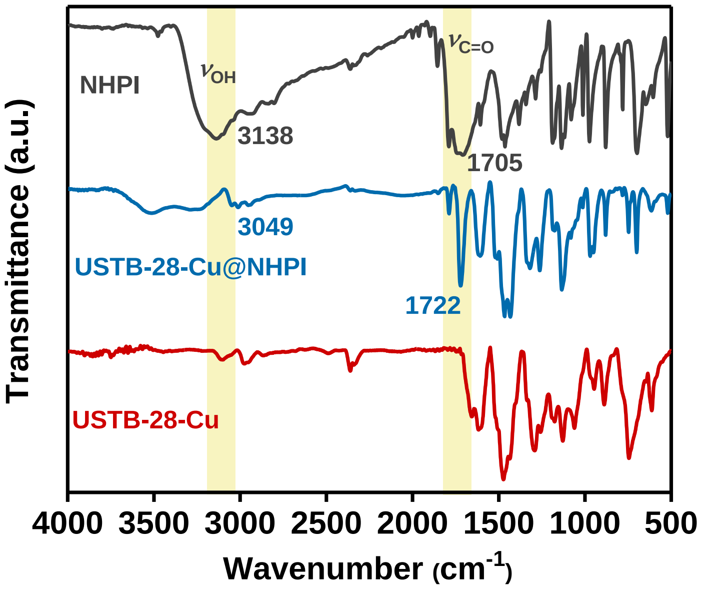

Figure S35. FT-IR spectra of NHPI, USTB-28-Cu@NHPI, and USTB-28-Cu.

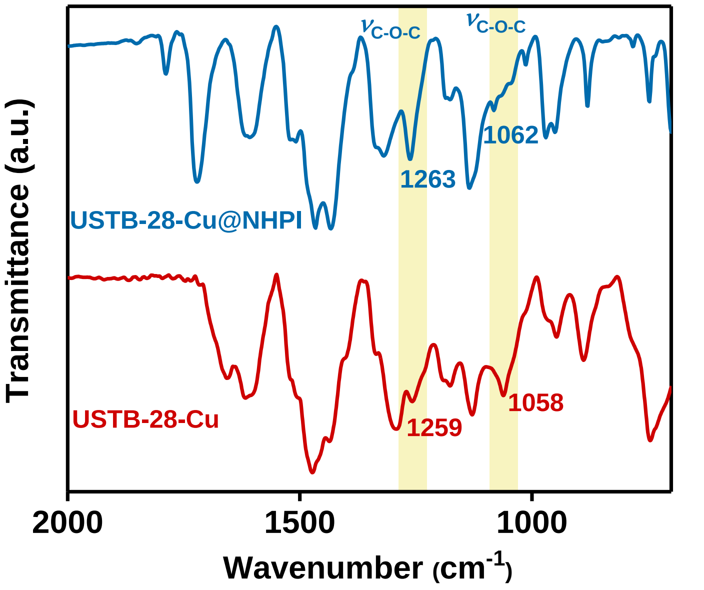

Figure S36. FT-IR spectra of USTB-28-Cu@NHPI, and USTB-28-Cu.

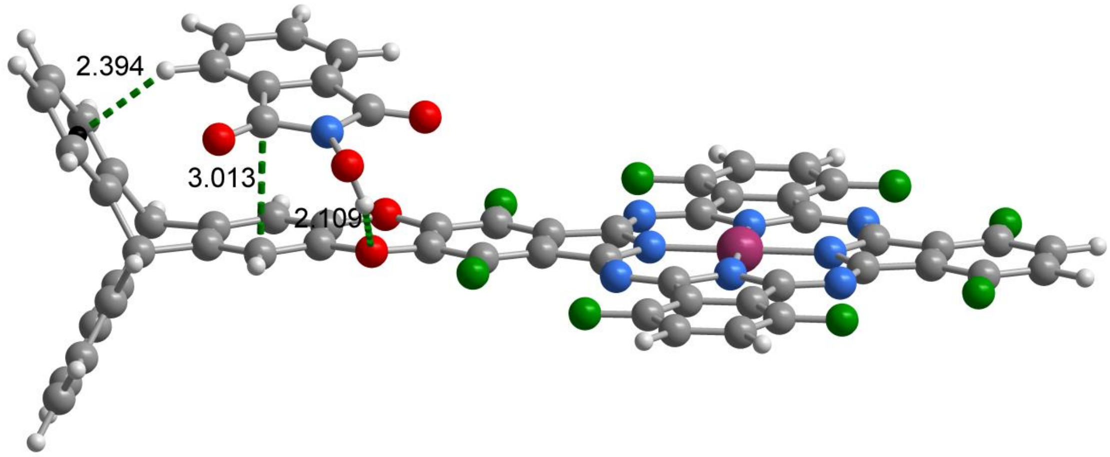

Figure S37. The different interaction between a simulated fragment from USTB-28-Co and NHPI.

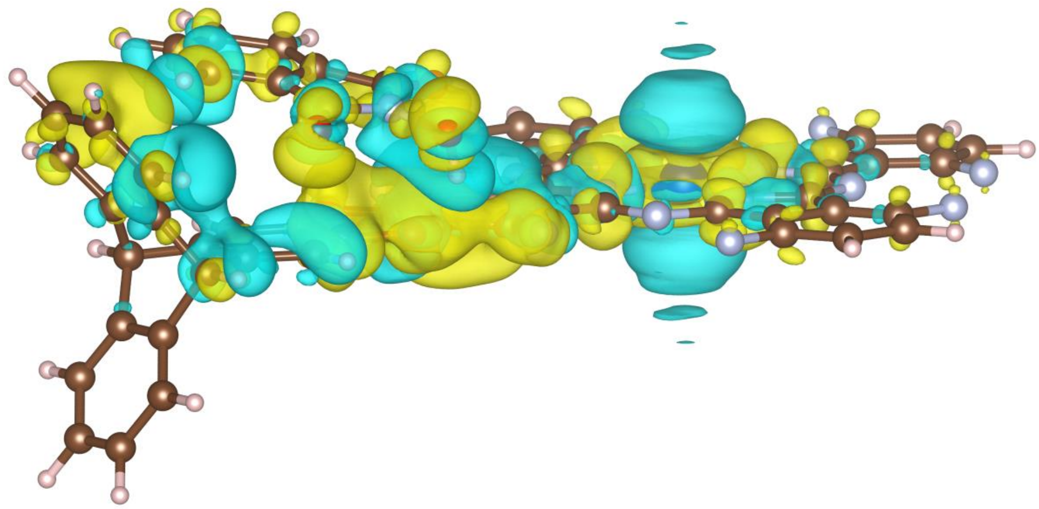

Figure S38. The charge density differences of a simulated fragment from USTB-28-Co interacting with NHPI. The yellow and cyan areas represented electron increase and decrease.

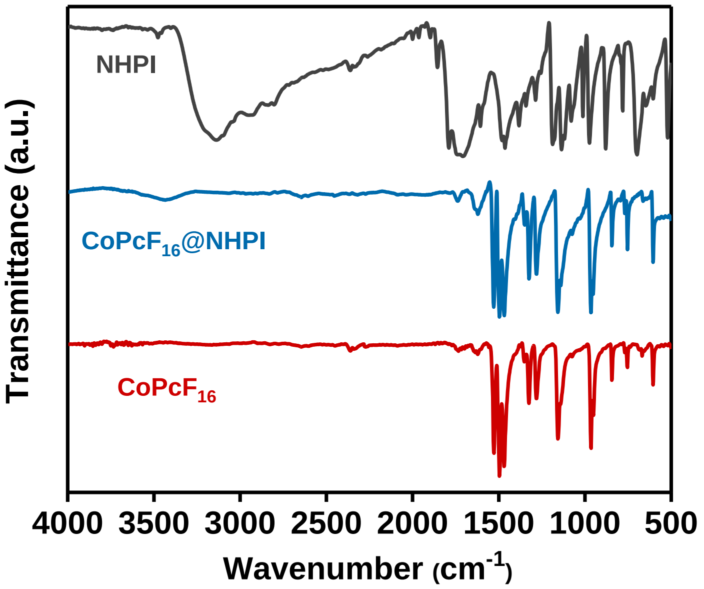

Figure S39. FT-IR spectra of the NHPI, $CoPcF_{16}$@NHPI, and $CoPcF_{16}$.

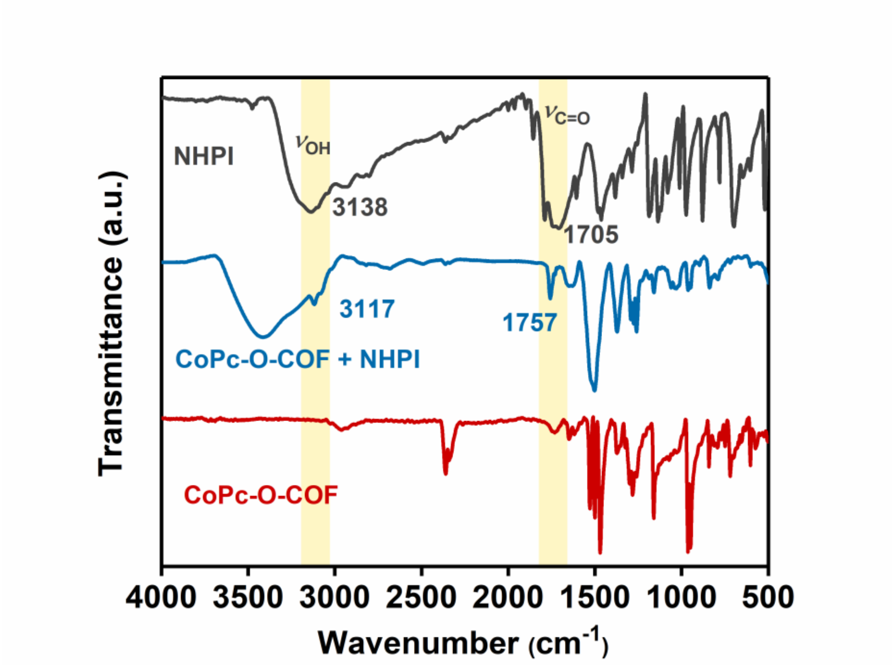

Figure S40. FT-IR spectra of the NHPI, CoPc-O-COF + NHPI, and CoPc-O-COF.

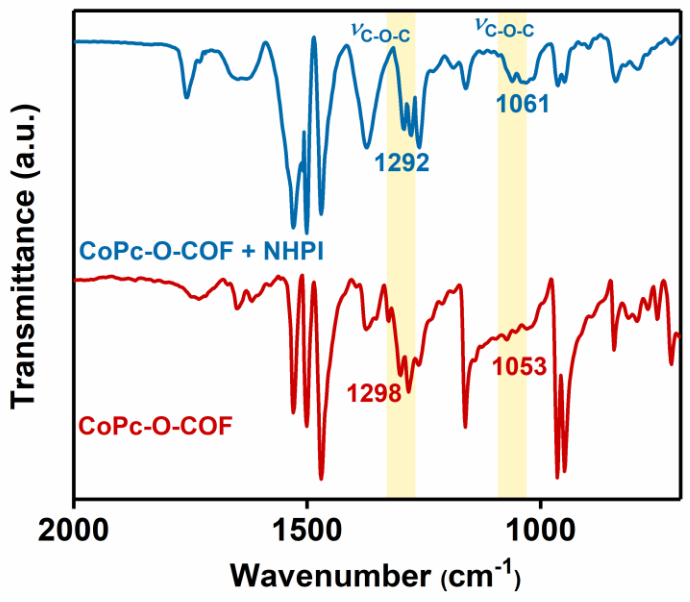

Figure S41. FT-IR spectra of the CoPc-O-COF + NHPI, and CoPc-O-COF.

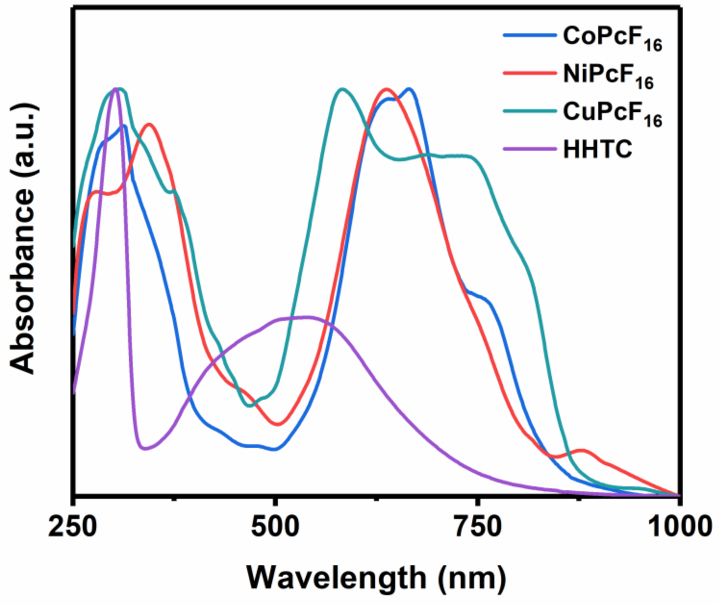

Figure S42. UV−vis-DRS of $CoPcF_{16}$, $NiPcF_{16}$, $CuPcF_{16}$, and HHTC, respectively.

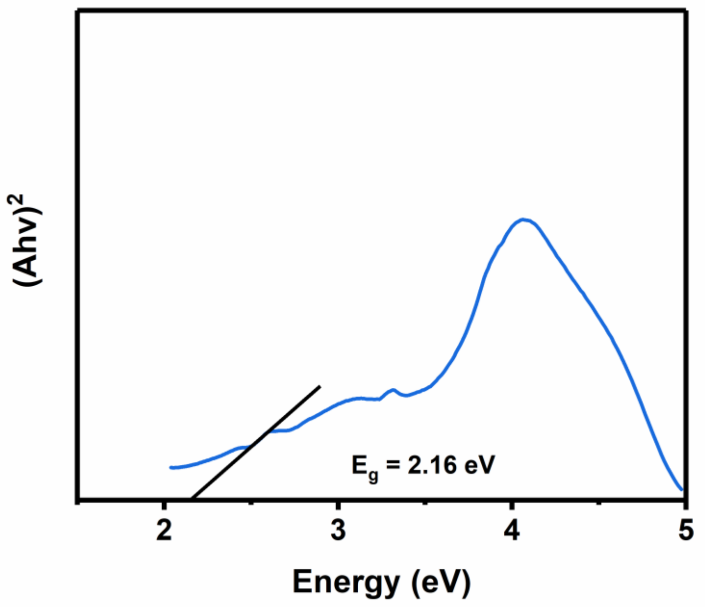

Figure S43. The value of band gap for USTB-28-Co.

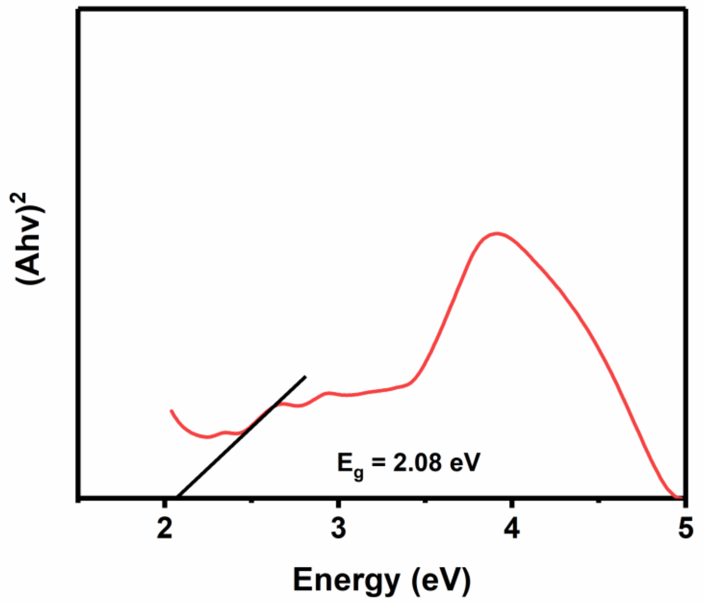

Figure S44. The value of band gap for USTB-28-Ni.

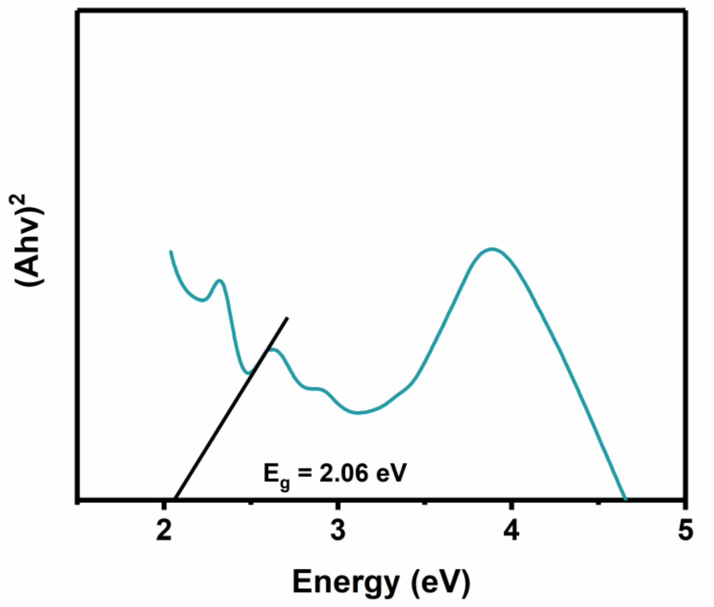

Figure S45. The value of band gap for USTB-28-Cu.

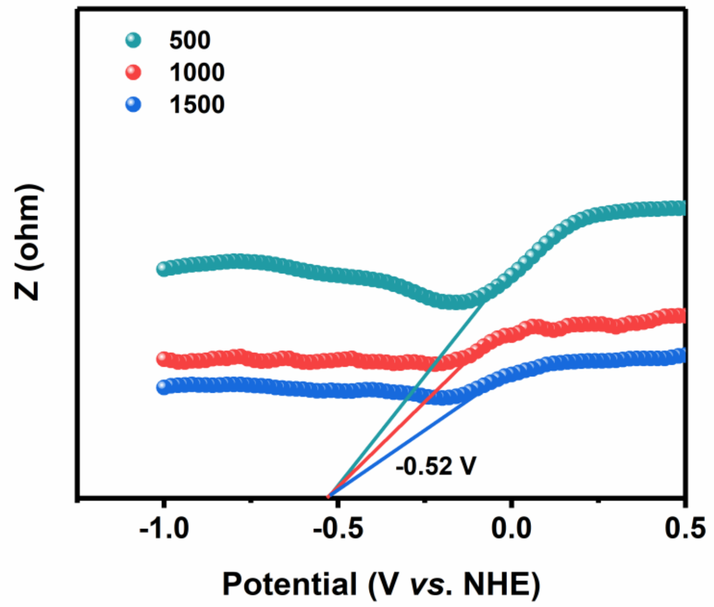

Figure S46. MS curves of USTB-28-Co.

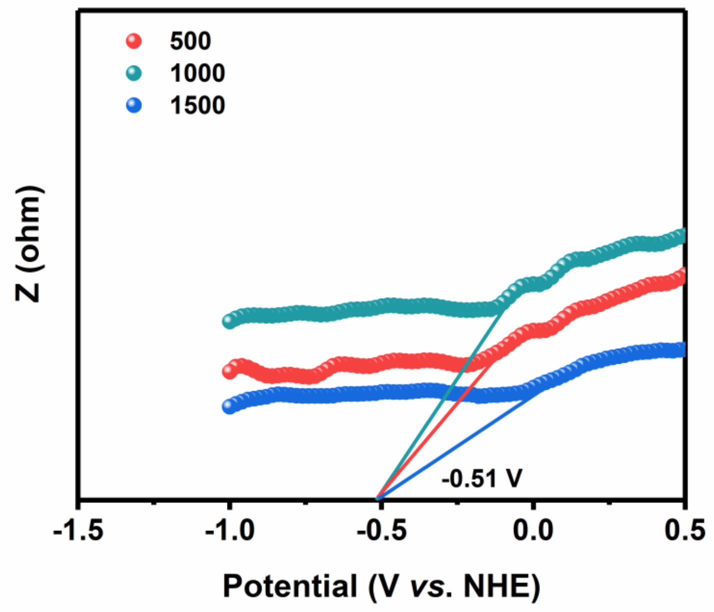

Figure S47. MS curves of USTB-28-Ni.

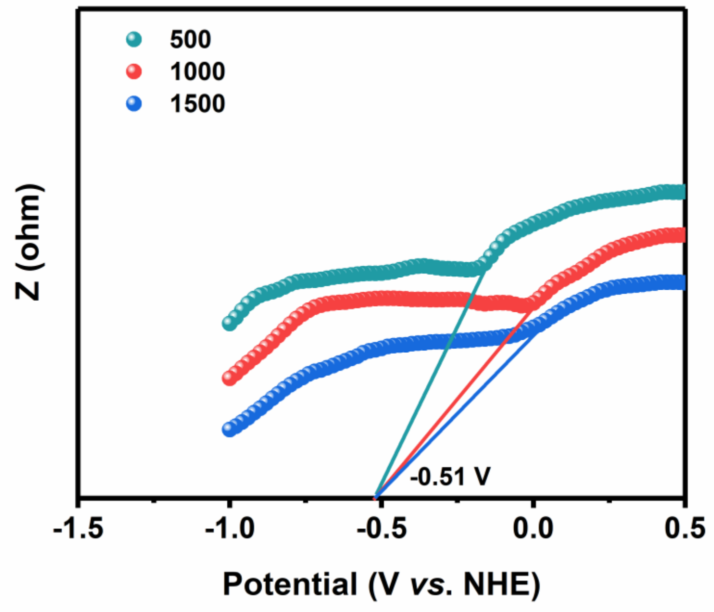

Figure S48. MS curves of USTB-28-Cu.

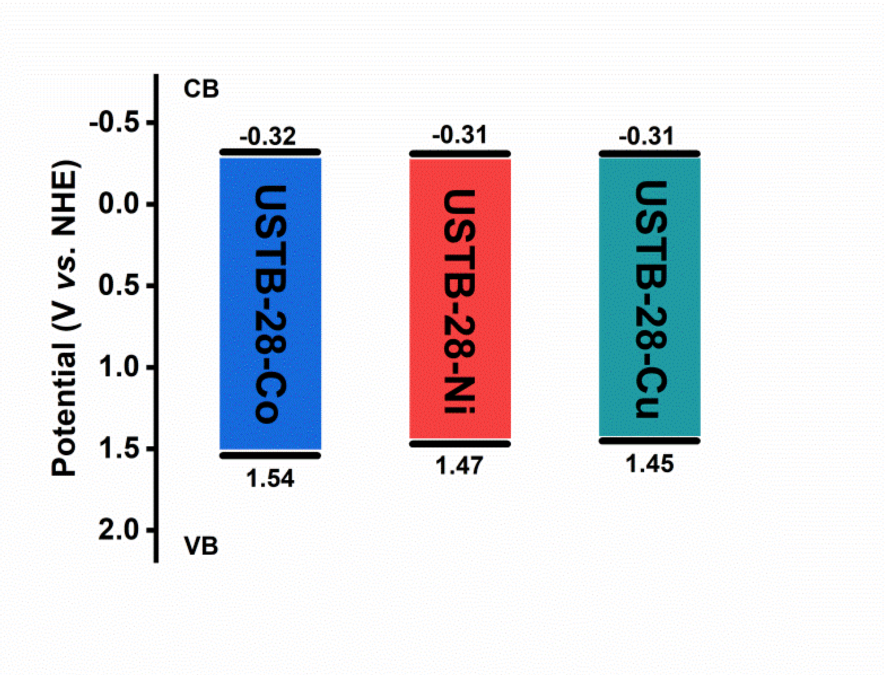

Figure S49. Band gaps of USTB-28-Co, USTB-28-Ni, and USTB-28-Cu, respectively.

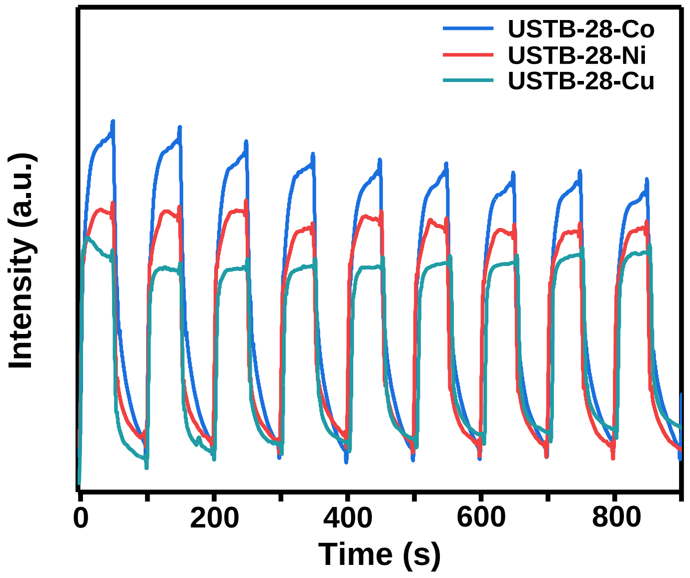

Figure S50. Photocurrents under Xe lamp irradiation of USTB-28-Co, USTB-28-Ni, and USTB-28-Cu, respectively.

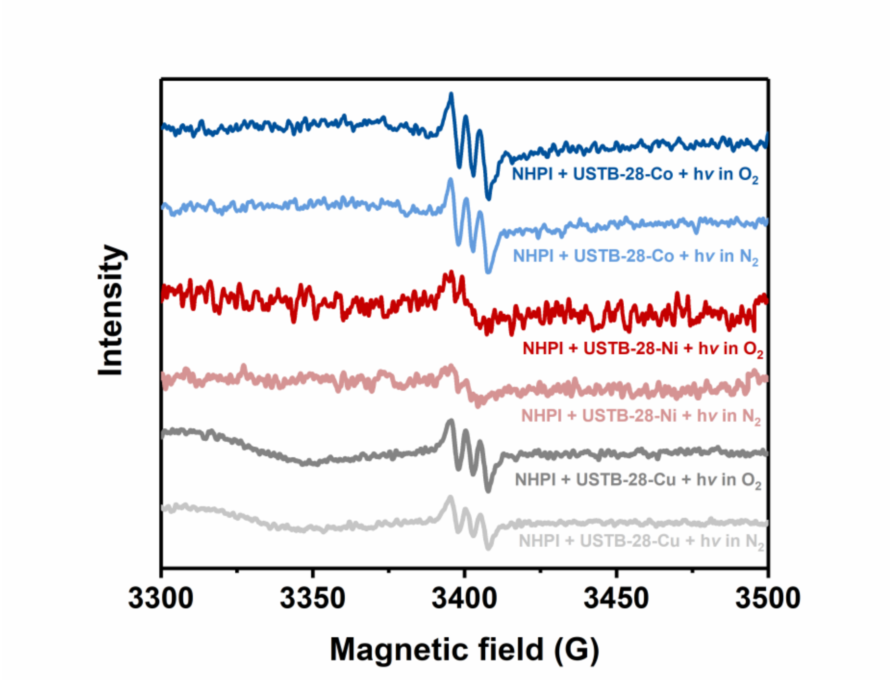

Figure S51. EPR data under the irradiation of a 350 W Xe lamp at O_2 and N_2 atmospheres.

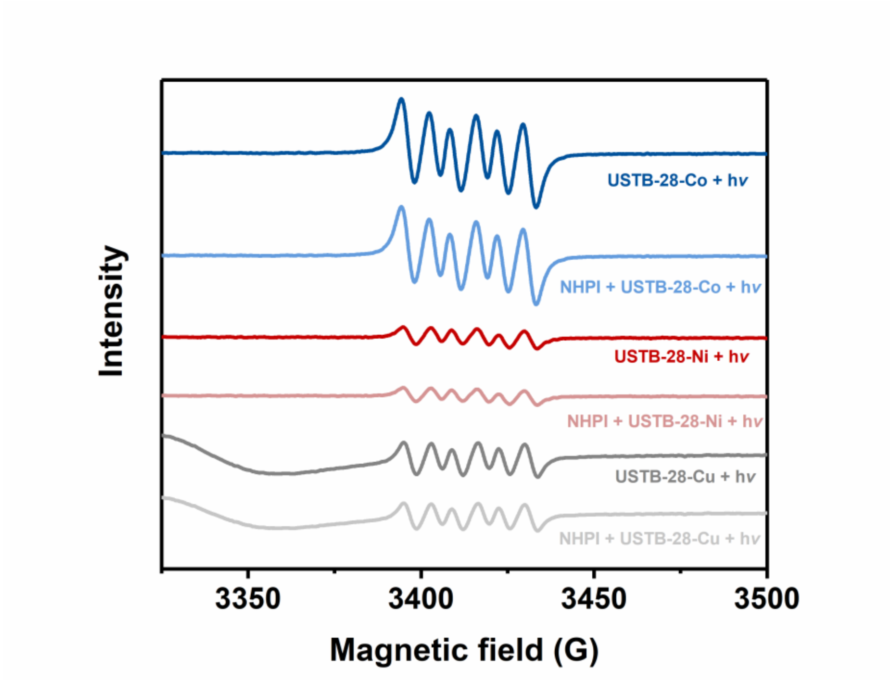

Figure S52. EPR EPR data under the irradiation of a 350 W Xe lamp in the presence of 5,5-dimethyl-1-pyrroline-*N*-oxide (DMPO).

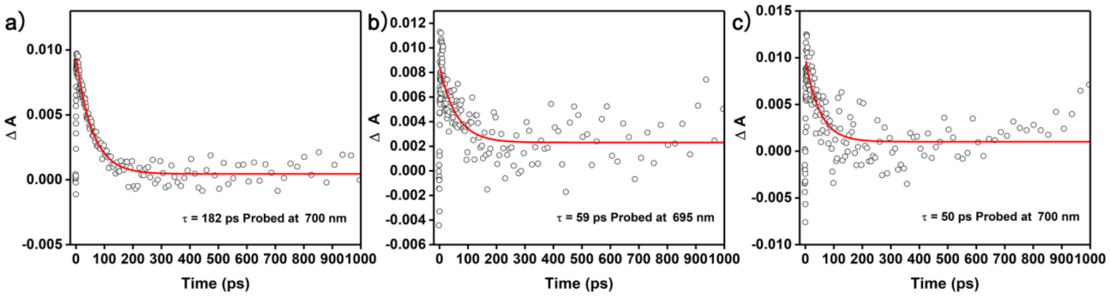

Figure S53. The kinetic trace of the (a) USTB-28-Co at 700 nm, (b) $CoPcF_{16}$ at 695 nm, and (c) CoPc-O-COF at 700 nm.

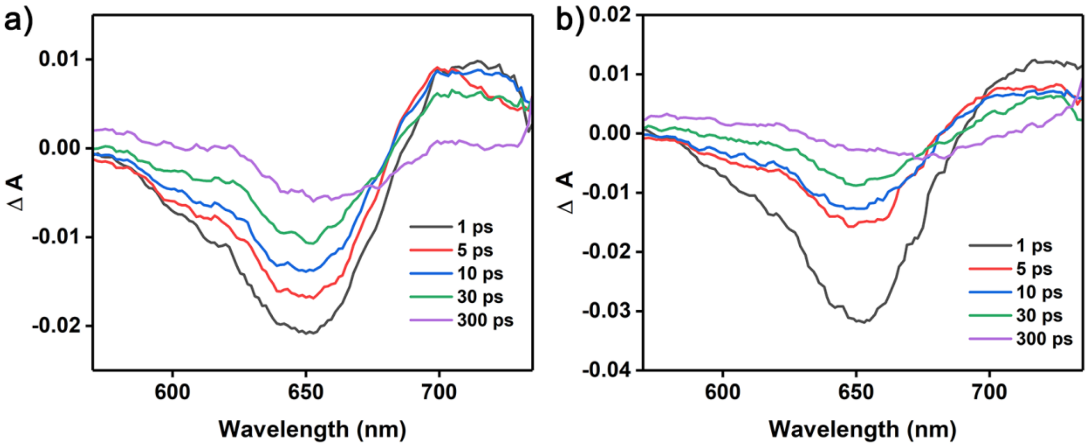

Figure S54. (a) The fs-TA spectra of the USTB-28-Co in O_2 atmosphere excited by 500 nm laser. (b) The fs-TA spectra of the USTB-28-Co + NHPI in O_2 atmosphere excited by 500 nm laser.

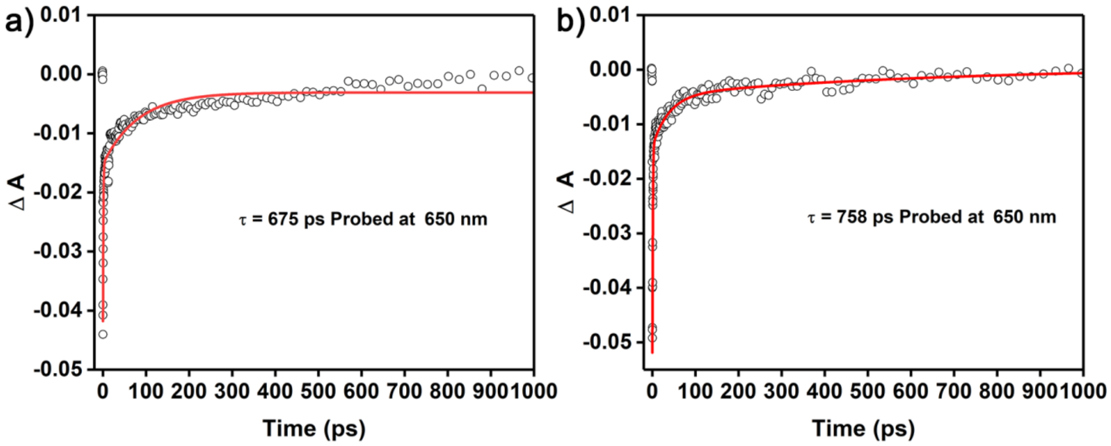

Figure S55. The kinetic trace of the (a) USTB-28-Co in O_2 atmosphere at 650 nm and (b) USTB-28-Co + NHPI in O_2 atmosphere at 650 nm.

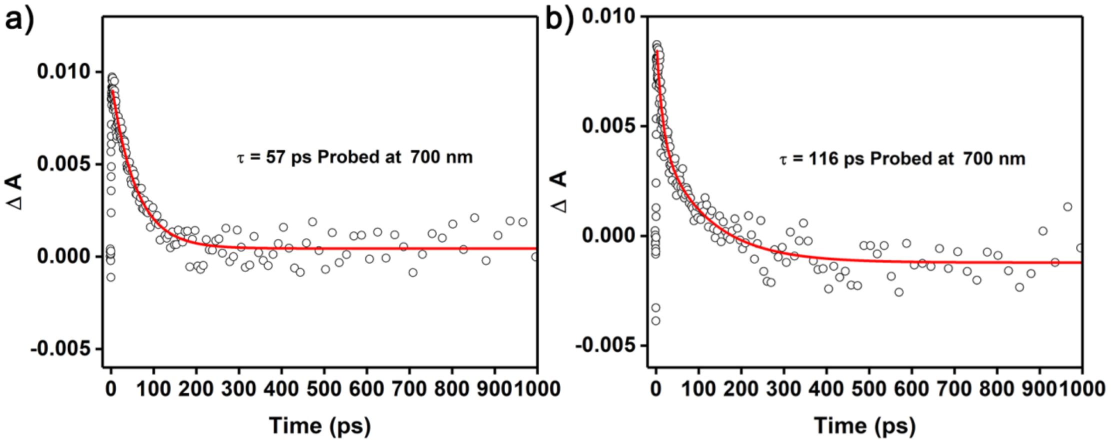

Figure S56. The kinetic trace of the (a) USTB-28-Co in O_2 atmosphere at 700 nm and (b) USTB-28-Co + NHPI in O_2 atmosphere at 700 nm.

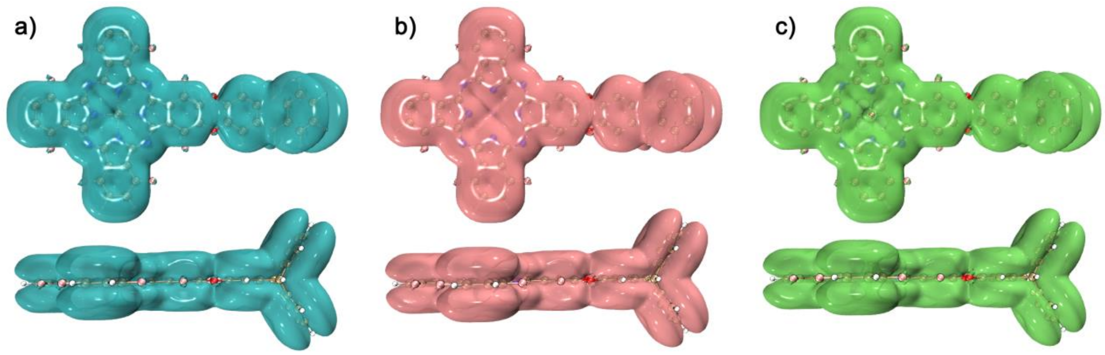

Figure S57. π-LOL of (a) USTB-28-Co, (b) USTB-28-Ni, and (c) USTB-28-Cu, respectively.

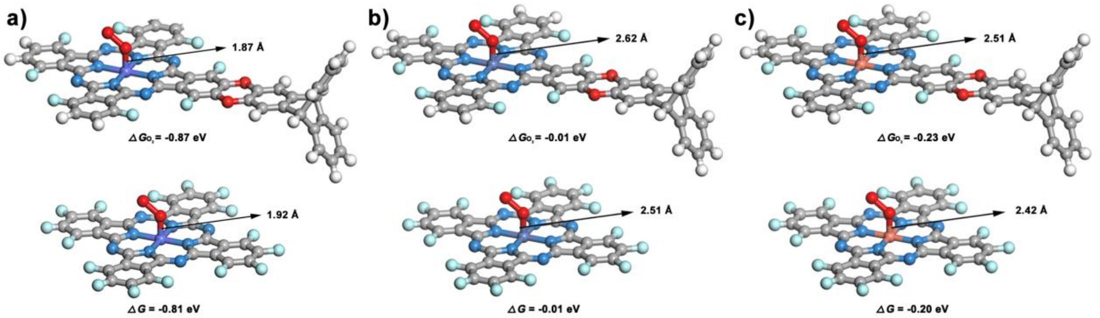

Figure S58. (a) Calculated oxygen adsorption energies on the Co atom of USTB-28-Co and $CoPcF_{16}$. (b) Calculated oxygen adsorption energies on the Ni atom of USTB-28-Ni and $NiPcF_{16}$. (c) Calculated oxygen adsorption energies on the Cu atom of USTB-28-Cu and $CuPcF_{16}$.

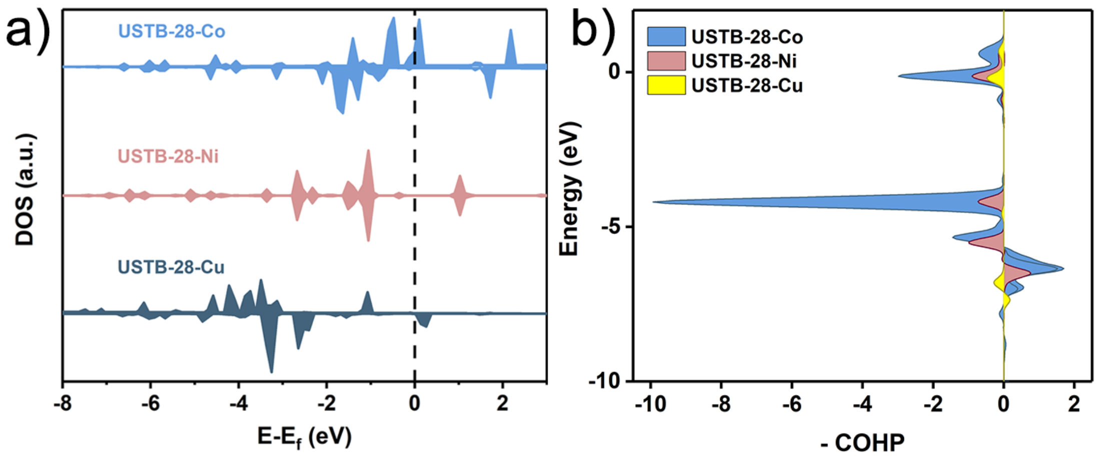

Figure S59. (A) The density of states of the metal atoms in USTB-28-M (M = Co, Ni, Cu). (B) The pCOHP analysis of active metal sites and O atoms from reactants USTB-28-M (M = Co, Ni, Cu).

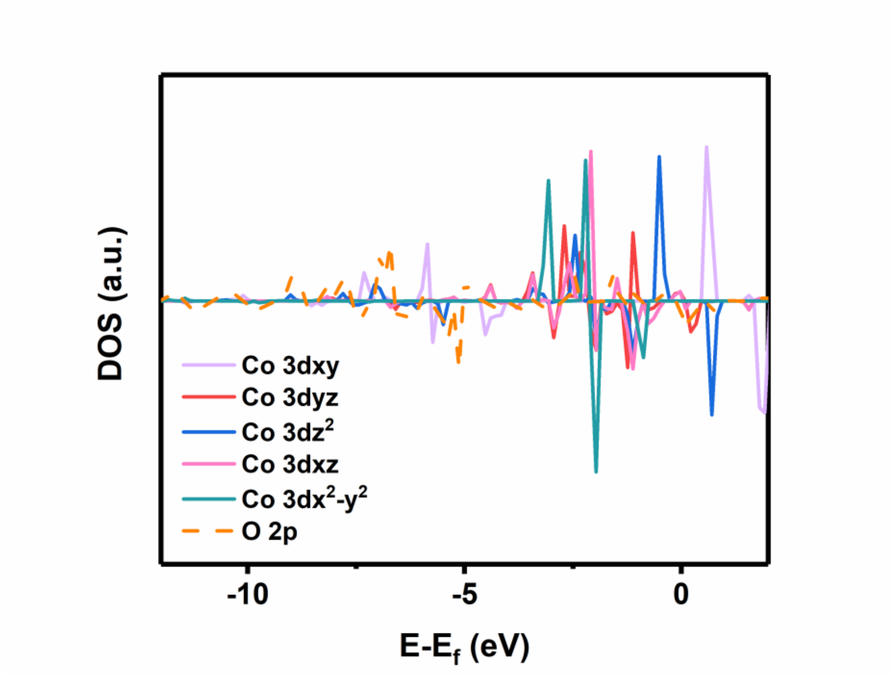

Figure S60. Projected density of states (pDOS) plots of 2p orbitals for O_2 and 3d orbitals (3dxy, 3dyz, 3dz^2 3dxz, 3dx^2-y^2) for USTB-28-Co.

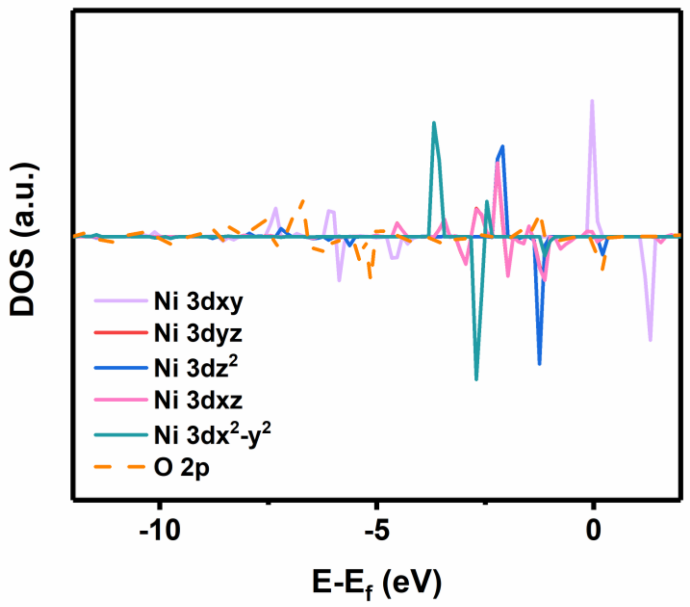

Figure S61. Projected density of states (pDOS) plots of 2p orbitals for O_2 and 3d orbitals ($3d_{xy}$, $3d_{yz}$, $3d_{z2}$ $3d_{xz}$, $3d_{x2-y2}$) for USTB-28-Ni.

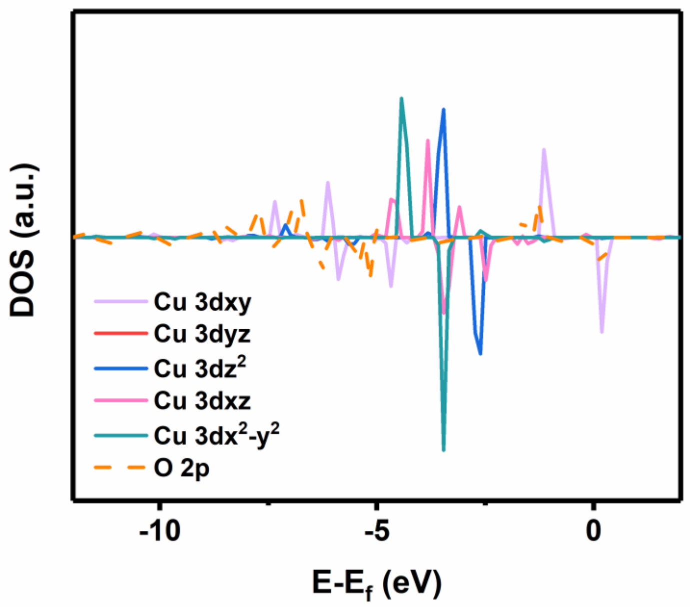

Figure S62. Projected density of states (pDOS) plots of 2p orbitals for O_2 and 3d orbitals ($3d_{xy}$, $3d_{yz}$, $3d_{z2}$ $3d_{xz}$, $3d_{x2-y2}$) for USTB-28-Cu.

Table S1. Synthetic conditions screened for the synthesis of USTB-28-Co.

**Entry**	**Solvent 1**	**Solvent 2**	**Base**	**Temperature**	**Days**	**Crystallinity**
**1**	1.0 mL DMF	---	0.1 mL Et_3N	120 ^oC	7	low
**2**	1.0 mL DMF	---	0.2 mL Et_3N	120 ^oC	7	low
**3**	2.0 mL DMF	---	0.2 mL Et_3N	120 ^oC	7	low
**4**	1.0 mL 1,4-dioxane	---	0.1 mL Et_3N	120 ^oC	7	low
**5**	1.0 mL DMAc	---	0.1 mL Et_3N	120 ^oC	7	amorphous
**6**	1.0 mL DMAc	---	0.2 mL Et_3N	120 ^oC	7	amorphous
**7**	1.0 mL NMP	---	0.1 mL Et_3N	120 ^oC	7	amorphous
**8**	1.0 mL NMP	---	0.2 mL Et_3N	120 ^oC	7	low
**9**	0.5 mL DMAc	0.5 mL Mes	0.2 mL Et_3N	120 ^oC	7	low
**10**	0.5 mL NMP	0.5 mL Mes	0.2 mL Et_3N	120 ^oC	7	low
**11**	0.65 mL NMP	0.35 mL Mes	0.2 mL Et_3N	120 ^oC	7	low
**12**	0.65 mL DMAc	0.35 mL Mes	0.2 mL Et_3N	120 ^oC	7	low
**13**	0.35 mL NMP	0.65 mL Mes	0.2 mL Et_3N	120 ^oC	7	high
**14**	0.35 mL DMAc	0.65 mL Mes	0.2 mL Et_3N	120 ^oC	7	low
**15**	0.35 mL NMP	0.65 mL Mes	0.2 mL Et_3N	100 ^oC	7	low
**16**	0.35 mL DMAc	0.65 mL Mes	0.2 mL Et_3N	100 ^oC	7	low
**17**	0.35 mL NMP	0.65 mL Mes	0.2 mL Et_3N	180 ^oC	7	high
**18**	0.35 mL DMAc	0.65 mL Mes	0.2 mL Et_3N	180 ^oC	7	low

Table S2. The fitting parameters for EXAFS data for USTB-28-Co, USTB-28-Ni, and USTB-28-Cu. (CN, coordination number; R, distance between absorber and backscatter atoms; σ^2 , Debye-Waller factor (a measure of thermal and static disorder in absorber-scatter distances); *R* factor is used to value the goodness of the fitting).

Sample	Path	CN	R (Å)	σ^2(10^{-3} Å^2)	*R* factor
USTB-28-Co	Co-N	4^f	1.99	1.08	0.01
USTB-28-Ni	Ni-N	4^f	2.01	7.73	0.01
USTB-28-Cu	Cu-N	4^f	2.06	6.15	0.01

Table S3. The tested ICP-OES results and calculated values of Co, Ni, and Cu for USTB-28-Co, USTB-28-Ni, and USTB-28-Cu, respectively

Sample	Calculated value	Tested value
USTB-28-Co	5.05%	4.91%
USTB-28-Ni	5.03%	4.92%
USTB-28-Cu	5.43%	5.24%

Table S4. Elemental analysis of USTB-28-Co, USTB-28-Ni, and USTB-28-Cu, respectively

Sample	Calculated Value (C, H, N)	Tested Value (C, H, N)
USTB-28-Co	60.41, 0.92, 9.61	58.06, 1.27, 9.23
USTB-28-Ni	60.43, 0.92, 9.61	58.36, 1.22, 9.28
USTB-28-Cu	60.18, 0.92, 9.57	58.94, 1.12, 9.37

Table S5. Photocatalytic oxidation of benzylic sp^3 C–H bonds of ethylbenzene with different Solvents. [a]

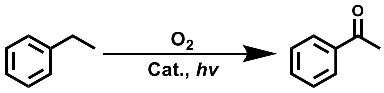

Entry	Solvent	Time /h	Yield %
1	Acetonitrile	2.0	59
2	Methanol	2.0	39
3	Ethanol	2.0	22
4	Ethyl acetate	2.0	15

[a] Reaction conditions: substrate (0.3 mmol), USTB-28-Co (0.002 mmol) and *N*-hydroxyphthalimide (0.02 mmol), Solvents (2.0 mL), O_2 (1 balloon), irradiated by a 350 W Xe lamp. [b] The yields of products were determined by gas chromatography (GC) areas with external standards, all results are yield.

Table S6. Photocatalytic oxidation of benzylic sp^3 C–H bonds of ethylbenzene with different catalysts to calculated TOF. [a]

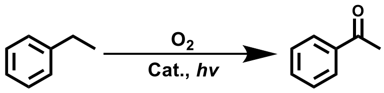

Entry	Catalyst	Yield (%)[b]	TOF
1	USTB-28-Co	42	63
2	USTB-28-Ni	22	33
3	USTB-28-Cu	30	45
4	$CoPcF_{16}$	5	8
5	$NiPcF_{16}$	3	5
6	$CuPcF_{16}$	4	6

[a] Reaction conditions: substrate (0.3 mmol), USTB-28-M or $MPcF_{16}$ (0.002 mmol based on MPc, M = Co, Ni, Cu) and *N*-hydroxyphthalimide (0.02 mmol), acetonitrile (2.0 mL), O_2 (1 balloon), irradiated by a 350 W Xe lamp for 1 h.
[b] The yields of products were determined by GC areas with an internal standard of naphthalene.

Table S7. Photocatalytic oxidation of benzylic sp^3 C–H bonds of ethylbenzene with different conditions. [a]

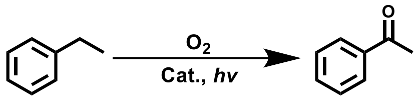

Entry	Catalyst	Yield (%)[b]	Select. (%)[b]	Conv. (%)[b]
1	USTB-28-Co	97	99	98
2[c]	USTB-28-Co	----	----	----
3	$CoPcF_{16}$	27	87	31
4[d]	----	----	----	----
5[e]	HHTC	----	----	----
6[f]	USTB-28-Co	97	99	98
7[g]	USTB-28-Co	96	98	98
8[h]	USTB-28-Co	94	97	97
9[i]	USTB-28-Co	93	99	94
10[j]	USTB-28-Co	93	98	95
11[k]	$CoPcF_{16}$	8	79	10
12[l]	$CoPcF_{16}$	----	----	12

[a] Reaction conditions: substrate (0.3 mmol), USTB-28-M or $CoPcF_{16}$ (0.002 mmol based on MPc, M = Co, Ni, Cu) and *N*-hydroxyphthalimide (0.02 mmol), acetonitrile (2.0 mL), O_2 (1 balloon), irradiated by a 350 W Xe lamp for 8 h.

[b] The yields of products were determined by GC areas with an internal standard of naphthalene.

[c] In the dark.

[d] No catalyst.

[e] HHTC (0.002 mmol)

[f] The second cycle.

[g] The third cycle.

[h] The fourth cycle.

[i] The fifth cycle.

[j] The sixth cycle.

[k] The second cycle.

[l] The third cycle.

Table S8. Comparison of catalytic performances for oxidation of ethylbenzene with various catalysts.

**Entry**	**Catalyst**	**TOF (h^{-1})**	**Reference**
1	USTB-28-Co	63.0	This work
2	$CoPcF_{16}$	8.0	This work
3	0.5%Pd@C-Gl_{UA}-550	71.0	S12
4	Co-ISA/CNB	57.8	S13
5	$Fe(TPA)[MeCN]_2(ClO_4)_2$	37.0	S14
6	Co-N-C	27.9	S15
7	Fe-N-C	24.7	S16
8	Co/AC	15.7	S17
9	CZJ-30	11.8	S18
10	CoCuAl-LDH/graphene	6.1	S19
11	Co/oxide	5.1	S20
12	Cu-BTC-SiO_2	3.3	S21
13	FTO|ZrO_2|FMN	3.2	S22
14	SACo@g-C_3N_4	2.3	S23
15	s-1	1.2	S24
16	ZJU-18	1.1	S25

3. References

1. Langis-Barsetti, S.; Maris, T.; Wuest, J. D. Triptycene 1,2-Quinones and Quinols: Permeable Crystalline Redox-Active Molecular Solids. *J. Org. Chem.* **2018**, *83*, 15426–15437.
2. Wu, J.; He, D.; Zhang, L.; Liu, Y.; Mo, X.; Lin, J.; Zhang, H. J. Direct synthesis of large-scale ortho-iodinated perylene diimides: key precursors for functional dyes. *Org. Lett.* **2017**, *19*, 5438–5441.
3. Kresse, G.; Furthmüller, J. Efficiency of Ab-initio Total Energy Calculations for Metals and Semiconductors Using A Plane-Wave Basis Set. *Comput. Mater. Sci*.**1996**, *6*, 15–50.
4. Kresse, G.; FurthmüLler, J. Efficient Iterative Schemes For Ab Initio Total-Energy Calculations Using A Plane-Wave Basis Set. *Phys. Rev. B: Condens. Matter Mater. Phys.* **1996**, *54*, 11169–11186.
5. Blochl, P. E. Projector Augmented-Wave Method. *Phys. Rev. B* **1994**, *50*, 17953–17979.
6. Perdew, J. P.; Burke, K.; Ernzerhof, M. Generalized Gradient Approximation Made Simple. *Phys. Rev. Lett.* **1997**, *77*, 3865–3868.
7. Delley, B. J. An All-Electron Numerical Method For Solving The Local Density Functional For Polyatomic Molecules. *Chem. Phys.* **1990**, *92*, 508– 517.
8. Gaussian 16, Revision B.01, Frisch, M. J.; Trucks G. W.; Schlegel, H. B.; Scuseria, G. E.; Robb, M. A.; Cheeseman, J. R.; Scalmani, G.; Barone, V.; Petersson, G. A.; Nakatsuji, H.; Li, X.; Caricato, M.; Marenich, Bloino, A. V. J.; Janesko, B. G.; Gomperts, R.; Mennucci, B.; Hratchian, H. P.; Ortiz, J. V.; Izmaylov, A. F.; Sonnenberg, J. L.; Williams-Young, D.; Ding, F.; Lipparini, F.; Egidi, F.; Goings, J.; Peng, B.; Petrone, A.; Henderson, T.; Ranasinghe, D.; Zakrzewski, V. G.; Gao, J.; Rega, N.; Zheng, G.; Liang, W.; Hada, M.; Ehara, M.; Toyota, K.; Fukuda, R.; Hasegawa, J.; Ishida, M.; Nakajima, T.; Honda, Y.; Kitao, O.; Nakai, H.; Vreven, T.; Throssell, K.; Montgomery, J. A.; Peralta, Jr., J. E.; Ogliaro, F.; Bearpark, M. J.; Heyd, J. J.; Brothers, E. N.; Kudin, K. N.; Staroverov, V. N.; Keith, T. A.; Kobayashi, R.; Normand, J.; Raghavachari, K.; Rendell, A. P.; Burant, J. C.; Iyengar, S. S.; Tomasi, J.; Cossi, M.; Millam, J. M.; Klene, M.; Adamo, C.; Cammi, R.; Ochterski, J. W.; Martin, R. L.; Morokuma, K.; Farkas, O.; Foresman, J. B.; and Fox, D. J.; Gaussian, Inc., Wallingford CT, **2016**.
9. Krishnan, R.; Binkley, J. S.; Seeger, R.; Pople, J. A. Self-consistent molecular orbital methods. XX. A basis set for correlated wave functions. *J. Chem. Phys.* **2008**, *72*, 650–654.
10. McLean, A. D.; Chandler, G. S. Contracted Gaussian basis sets for molecular calculations. I. Second row atoms, Z=11–18. *J. Chem. Phys.* **2008**, *72*, 5639–5648.
11. Zhao, Y.; Truhlar, D. G. The M06 suite of density functionals for main group thermochemistry, thermochemical kinetics, noncovalent interactions, excited states, and transition elements: two new functionals and systematic testing of four M06-class functionals and 12 other functionals. *Theor Chem Acc* **2008**, *120*, 215–241.

12. Zhang, P.; Gong, Y.; Li, H.; Chen, Z.; Wang, Y. Solvent-free aerobic oxidation of hydrocarbons and alcohols with Pd@N-doped carbon from glucose. *Nat. Commun.* **2013**, *4*, 1593.
13. Zhu, Y.; Sun, W.; Chen, W.; Cao, T.; Xiong, Y.; Luo, J.; Dong, J.; Zheng, L.; Zhang, J.; Wang, X.; Chen, C.; Peng, Q.; Wang, D.; Li, Y. Scale-Up Biomass Pathway to Cobalt Single-Site Catalysts Anchored on N-Doped Porous Carbon Nanobelt with Ultrahigh Surface Area. *Adv. Funct. Mater.* **2018**, *28*, 1802167.
14. Mühldorf, B.; Wolf, R. C–H Photooxygenation of Alkyl Benzenes Catalyzed by Riboflavin Tetraacetate and a Non-Heme Iron Catalyst. *Angew. Chem. Int. Ed.* **2016**, *55*, 427–430.
15. Chen, Y.; Jie, S.; Yang, C.; Liu, Z. Active and efficient Co-N/C catalysts derived from cobalt porphyrin for selective oxidation of alkylaromatics. *Appl. Surf. Sci.* **2017**, *419*, 98–106.
16. Liu, W.; Zhang, L.; Liu, X.; Liu, X.; Yang, X.; Miao, S.; Wang, W.; Wang, A.; Zhang, T. Discriminating Catalytically Active FeNx Species of Atomically Dispersed Fe–N–C Catalyst for Selective Oxidation of the C–H Bond. *J. Am. Chem. Soc.* **2017**, *139*, 10790–10798.
17. Nakatsuka, K.; Yoshii, T.; Kuwahara, Y.; Mori, K.; Yamashita, H. Controlled synthesis of carbon-supported Co catalysts from single-sites to nanoparticles: characterization of the structural transformation and investigation of their oxidation catalysis. *Physical Chemistry Chemical Physics* **2017**, *19*, 4967–4974.
18. He, W.-L.; Wu, C.-D. Incorporation of Fe-phthalocyanines into a porous organic framework for highly efficient photocatalytic oxidation of arylalkanes. *Appl. Catal. B* **2018**, *234*, 290–295.
19. Xie, R.; Fan, G.; Yang, L.; Li, F. Highly Efficient Hybrid Cobalt–Copper–Aluminum Layered Double Hydroxide/Graphene Nanocomposites as Catalysts for the Oxidation of Alkylaromatics. *ChemCatChem* **2016**, *8*, 363–371.
20. Xie, R.; Fan, G.; Yang, L.; Li, F. Solvent-free oxidation of ethylbenzene over hierarchical flower-like core–shell structured Co-based mixed metal oxides with significantly enhanced catalytic performance. *Catal. Sci. Technol.* **2015**, *5*, 540–548.
21. Song, G.-Q.; Lu, Y.-X.; Zhang, Q.; Wang, F.; Ma, X.-K.; Huang, X.-F.; Zhang, Z.-H. Porous Cu–BTC silica monoliths as efficient heterogeneous catalysts for the selective oxidation of alkylbenzenes. *RSC Adv.* **2014**, *4*, 30221–30224.
22. Dongare, P.; MacKenzie, I.; Wang, D.; Nicewicz, D. A.; Meyer, T. J. Oxidation of alkyl benzenes by a flavin photooxidation catalyst on nanostructured metal-oxide films. *PNAS.* **2017**, *114*, 9279–9283.
23. Li, J.; Zhao, S.; Yang, S.-Z.; Wang, S.; Sun, H.; Jiang, S. P.; Johannessen, B.; Liu, S. Atomically dispersed cobalt on graphitic carbon nitride as a robust catalyst for selective oxidation of ethylbenzene by peroxymonosulfate. *J. Mater. Chem. A* **2021**, *9*, 3029–3035.
24. Cai, Y.; Zhang, J.-W.; Li, F.; Liu, J.-M.; Shi, S.-L. Nickel/N-Heterocyclic Carbene Complex-Catalyzed Enantioselective Redox-Neutral Coupling of Benzyl Alcohols and Alkynes to Allylic Alcohols. *ACS Catal.* **2019**, *9*, 1–6.

25. Yang, X.-L.; Xie, M.-H.; Zou, C.; He, Y.; Chen, B.; O'Keeffe, M.; Wu, C.-D. Porous Metalloporphyrinic Frameworks Constructed from Metal 5,10,15,20-Tetrakis(3,5-biscarboxylphenyl)porphyrin for Highly Efficient and Selective Catalytic Oxidation of Alkylbenzenes. *J. Am. Chem. Soc.* **2012**, *134*, 10638–10645.
